# Wired together, change together: Spike timing modifies transmission in converging assemblies

**DOI:** 10.1126/sciadv.adj4411

**Published:** 2024-01-17

**Authors:** Lidor Spivak, Shirly Someck, Amir Levi, Shir Sivroni, Eran Stark

**Affiliations:** ^1^ Sagol School of Neuroscience and Department of Physiology and Pharmacology, Faculty of Medicine, Tel Aviv University, Tel Aviv 6997801, Israel.; ^2^ Department of Mathematics, Afeka-Tel Aviv College of Engineering, Tel-Aviv 6910717, Israel.; ^3^ Department of Mathematics, The Open University of Israel, Ra’anana 4353701, Israel.; ^4^ Sagol Department of Neurobiology, Faculty of Natural Sciences, Haifa University, Haifa 3103301, Israel.

## Abstract

The precise timing of neuronal spikes may lead to changes in synaptic connectivity and is thought to be crucial for learning and memory. However, the effect of spike timing on neuronal connectivity in the intact brain remains unknown. Using closed-loop optogenetic stimulation in CA1 of freely moving mice, we generated unique spike patterns between presynaptic pyramidal cells (PYRs) and postsynaptic parvalbumin (PV)–immunoreactive cells. The stimulation led to spike transmission changes that occurred together across all presynaptic PYRs connected to the same postsynaptic PV cell. The precise timing of all presynaptic and postsynaptic cell spikes affected transmission changes. These findings reveal an unexpected plasticity mechanism, in which the spike timing of an entire cell assembly has a more substantial impact on effective connectivity than that of individual cell pairs.

## Teaser

Closed-loop optogenetics shows that the spike timing of cell assemblies affects spike transmission, exhibiting concurrent plasticity.

## INTRODUCTION

At the core of our capability for learning and memory is the capacity of the brain to adapt and modify in accordance with external events (*1*, *2*). Learning is supported by changes in synaptic connections between neurons, modulated by different plasticity rules (*3*, *4*). One model, spike timing–dependent plasticity (STDP), posits that changes in synaptic connectivity are driven by the relative timing of spikes between pre- and postsynaptic neurons (*5*–*8*). In vitro experiments showed that the millisecond timescale of spike timing between a pair of neurons influences their synaptic connectivity (*9*–*12*). However, the experiments did not reveal whether similar plasticity rules apply in the intact brain, where numerous cells are active simultaneously.

STDP studies in intact animals typically involved pairing the activity of a single postsynaptic cell with either sensory (*13*–*15*) or optogenetic stimuli (*16*, *17*). However, external stimuli activate an entire presynaptic pool rather than a single presynaptic neuron as done in vitro. In addition, these studies assessed plasticity changes based on the response of the postsynaptic cell to external stimuli, neglecting spike timing and alterations of individual connections between the cells. Thus, the impact of the spike timing of individual pairs within the same assembly on their connectivity remains unclear.

**Fig. 1.**
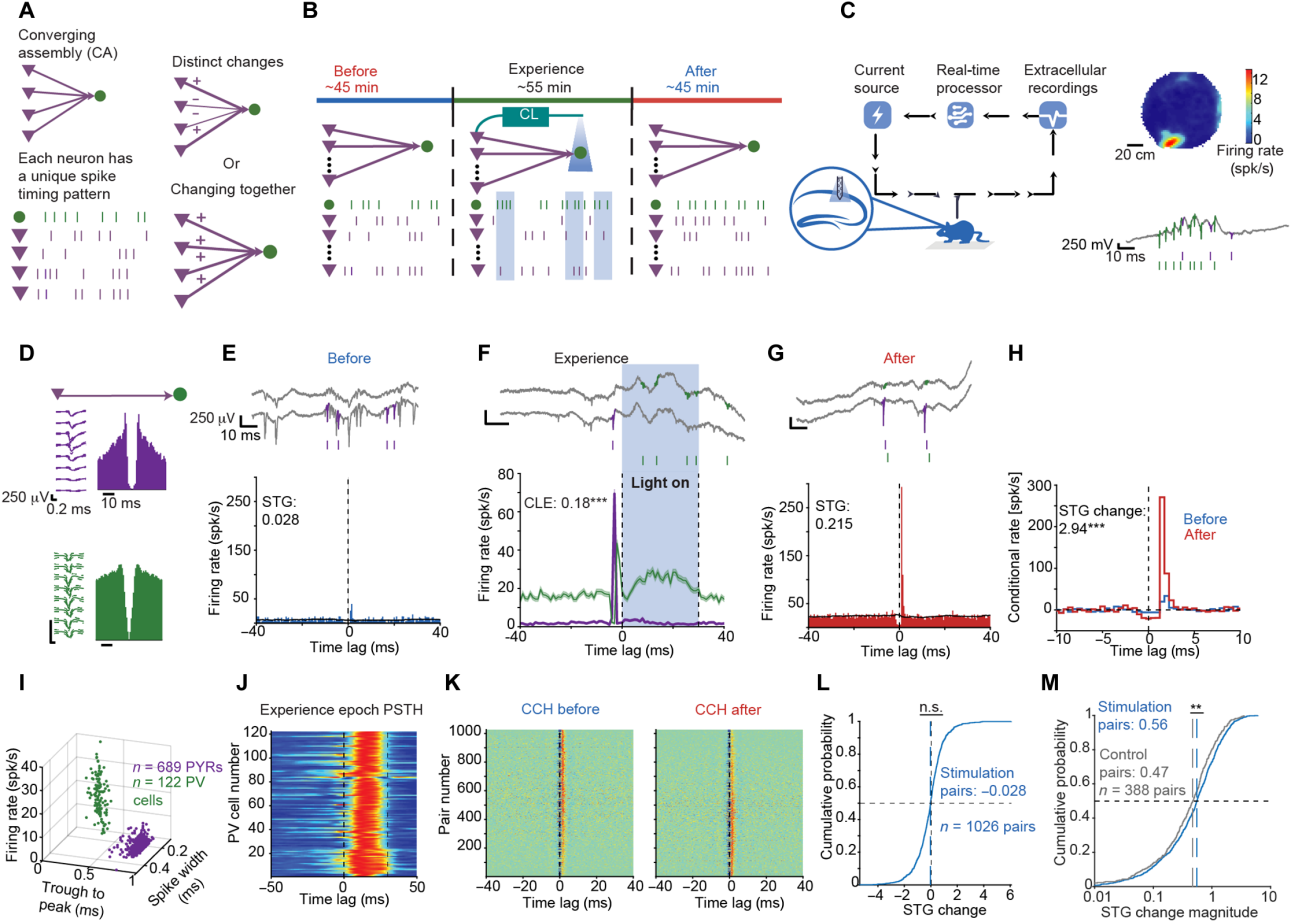
Changes in spike transmission gain occur after closed-loop induction of PV spikes. (**A**) The spike transmission gain (STG) between two neurons in a CA may be affected by their spike timing, spike timing of the entire CA, or be spike timing history–independent. (**B**) Experimental paradigm. (**C**) Closed-loop system. Left: During the Experience epoch, spikes of one or more PYRs detected in real-time generate light-induced spiking in nearby PV cells. Right: Place field of a PYR on the open field, and wideband (0.1 to 7 500 Hz) trace during a ripple event. (**D**) Wideband waveforms and autocorrelation histograms of a pre-/postsynaptic PYR-PV pair. (**E**) Conditional rate cross-correlation histogram (CCH) during the Before epoch. Top: Wideband traces. (**F**) Experience epoch peristimulus time histograms (PSTHs). PV firing rate increases immediately after PYR spiking, and again during the light. CLE, closed-loop efficiency. (**G**) After epoch CCHs. (**H**) Overlaid conditional rate CCHs for the no-light epochs obtained by removing the baseline activity [(E) and (F), black]. (**I**) Features of PYRs and PV cells recorded during stimulation experiments. (**J**) PSTHs of the PV cells. (**K**) All pairwise CCHs exhibiting excitatory connectivity. (**L**) STG changes for the Stimulation PYR-PV pairs. n.s., *P* > 0.05, Wilcoxon’s test. (**M**) Absolute STG changes for Stimulation and Control pairs. **P < 0.01, *U* test.

To investigate how spike timing affects connectivity in the intact brain when multiple neurons are involved, we recorded the simultaneous activity of dozens of pyramidal cells (PYRs) and parvalbumin (PV) interneurons in mouse CA1. The PV cells in the CA1 pyramidal cell layer are predominantly of basket cell morphology, receive excitatory input from multiple PYRs, and demonstrate a wide range of connection strengths (*18*–*21*). Together, a set of presynaptic PYRs and their postsynaptic target PV cell form a converging assembly (CA; Fig. 1A). The CA architecture makes the PYR-to-PV interface useful for testing how spike timing changes neuronal connectivity with respect to other connections.

Among excitatory connections to CA1 basket cells, one possibility is that the effect of the spike timing of each pair is purely homosynaptic and synapse-specific, affecting its own connection independently of other connections within the same assembly (*16*, *22*). Alternatively, the spike timing of specific pairs in the assembly may exert heterosynaptic changes, affecting the connections of other pairs within the same assembly in the same direction (*23*, *24*). In other brain regions, changes in the opposite direction have been observed (*25*). Heterosynaptic plasticity of excitatory inputs to basket cells may be facilitated via compartmentalized changes in intracellular calcium (*26*, *27*) or via excitability-based (*28*–*31*) mechanisms.

## RESULTS

### Changes in spike transmission gain occur after closed-loop induction of PV spikes

To determine how spike timing between multiple presynaptic PYRs and a postsynaptic PV cell influences the effective connectivity, we designed the following experiment (Fig. 1B). We recorded baseline CA1 network activity for a median of 45 min (Before epoch), followed by 55 min of “Experience” and an additional 45 min of baseline (“After”). During all three epochs, mice were free to behave in a familiar environment (table S1; Fig. 1C). This experimental paradigm enabled manipulating PYR-PV spike timing during the Experience epoch only and measuring the effects of the alternated spike timing on the changes in PYR-PV effective connectivity between the Before and After epochs. To manipulate spike timing, we implanted multi-shank diode probes in hippocampal region CA1 of four PV::ChR2 mice. Every probe was equipped with four diode-coupled optical fibers (fig. S1A and table S2) (*32*), enabling the activation of PV cells using optogenetic stimulation (Fig. 1C). For closed-loop optogenetic stimulation, a single spike of one or more PYRs was detected in real time (Fig. 1, D and F). After a 3-ms processing delay dedicated to sorting the spike, a 30-ms light stimulus was given, inducing spiking in nearby PV cells. The 30-ms light stimulation was sufficiently long for PV cell activation (Fig. 1J) but was too short to induce PYR spiking rebound (*33*) immediately after light termination (fig. S2). To quantify the effective connectivity, we used the spike transmission gain [STG; (*34*)] metric (Fig. 1, E to G). To assess long-term changes in STG between the Before and After epochs, we defined the “STG change” as the base-2 logarithm of the ratio between the STG_After_ and the STG_Before_ (Fig. 1H), where a change of 1 or −1 indicates STG doubling or halving following the Experience epoch.

To understand how changes in spike timing during the Experience epoch affect changes in spike transmission, we focused on PYR-PV pairs that exhibit monosynaptic connectivity (*P* < 0.001, Poisson test) and in which the PV cell (Fig. 1I) was activated by the closed-loop stimulation (*P* < 0.05, Poisson test; Fig. 1J). PYRs and PV cells could be accurately differentiated based on waveform features or spike timing statistics (Fig. 1I). A set of 689 connectivity-tagged PYRs and 122 optically tagged PV cells yielded a cohort of 1026 Stimulation pairs (Fig. 1K) recorded during a total of 29 sessions from the four freely moving PV::ChR2 mice (table S2). Among these pairs, the median STG change was not consistently different from zero (−0.028; *P* = 0.09; Wilcoxon’s test; Fig. 1L). Thus, consistent with work that quantified changes in PYR-interneuron connectivity during learning (*35*), there is an equilibrium of changes in STG at the population level.

To determine whether the observed changes exceed spontaneous changes, we compared the 1026 Stimulation pairs with Control pairs recorded during long no-stimulus periods from the CA1 of five mice (table S3). All 388 Control pairs exhibited monosynaptic connections (*P* < 0.001, Poisson test) but were not exposed to any light stimuli. The median STG change of the Control pairs (0.004) was not consistently different from the median STG of the Stimulation pairs (*P* = 0.37, *U* test). However, the magnitude of the STG changes among the Stimulation pairs (median [interquartile range IQR]: 0.556 [0.240 1.079]) was higher than Control pairs (0.473 [0.205 0.863]; *P* = 0.006, *U* test; Fig. 1M). Thus, while the overall net STG change remains balanced, the magnitude of STG changes increases following closed-loop stimulation during the Experience epoch.

### Changes in spike transmission occur together in a converging assembly

**Fig. 2.**
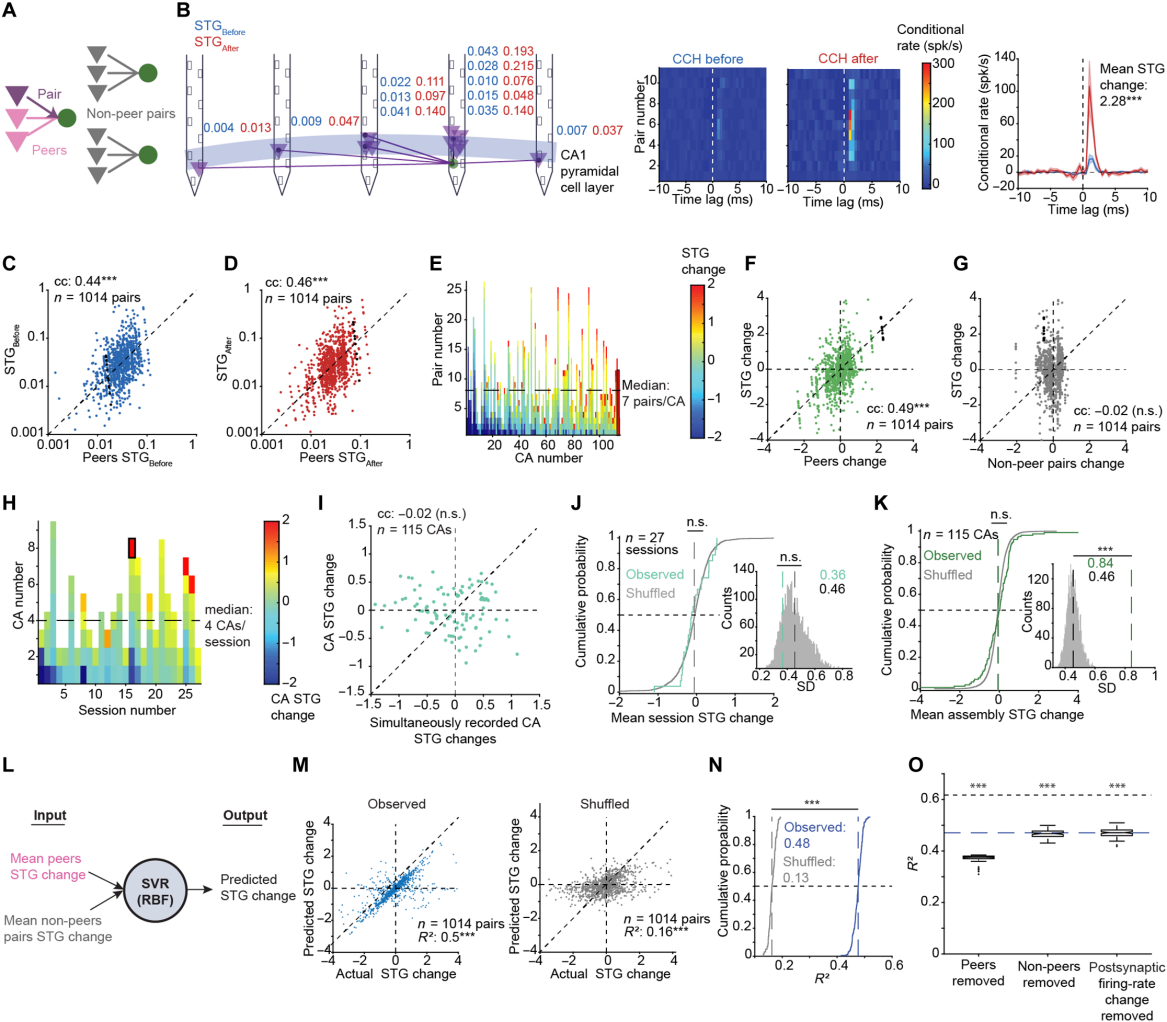
Changes in spike transmission occur together in a converging assembly. (**A**) Peer/non-peer nomenclature for simultaneous-recorded PYR-PV pairs based on the relation to a specific pair. (**B**) Example 12-unit CA, CCHs for the 11 pairs, and average CCHs. Here and in (C), (D), (F), (G), and (I) to (K), n.s., *P* > 0.05; ***P < 0.001, permutation test. (**C**) Pairwise STG_Before_ versus the mean peer STG_Before_. (**D**) Same, for STG_After_. (**E**) Pairwise STG changes per CA. (**F**) Pairwise STG change versus mean STG change of all peers. (**G**) Same, versus mean non-peer STG change. (**H**) CA STG changes per session. (**I**) CA STG change versus mean STG change of all other simultaneously recorded CAs. (**J**) Mean STG changes of same-session pairs and chance distribution. Right: SD of same-session STG changes. (**K**) Mean STG changes of all multi-pair CAs and chance distribution. Right: SD of CA STG changes. (**L**) Cross-validated SVR. (**M**) Left: Predicted versus actual STG changes. Right: Same, for intra-session shuffled allocation. Here and in (O), ***P < 0.001, Wilcoxon’s test. (**N**) *R*^2^ values derived from 100 independently generated SVRs. ***P < 0.001, *U* test. (**O**) Contribution of postsynaptic firing rate to STG change prediction. Every box plot shows the median and interquartile range (IQR), whiskers extend for ±1.5 IQRs, and individual dots indicate outliers.

To investigate STG equilibrium, we considered two scenarios. First, equilibrium is maintained by each CA, implying that net STG changes for every assembly are near zero. Second, equilibrium is maintained only at a higher level, where some CAs exhibit a net STG increase and others a decrease. To distinguish between the scenarios, we compared the STGs of individual pairs to other pairs within the same assembly, referred to as “peer pairs” (Fig. 2A). To account for more global changes including influences of behavior or brain state changes on STGs, we compared the STG of the individual pair to every PYR-PV pair within other simultaneously recorded CAs, referred to as “non-peer pairs” (Fig. 2A).

We found that during both the Before and After epochs, single-pair STG was correlated with the mean peers STG [Before: rank correlation coefficient (cc): 0.44; *P* < 0.001, permutation test; After: cc: 0.46; *P* < 0.001; Fig. 2, C and D]. The correlation between single-pair STG and peer STG was also observed in two other datasets, recorded from CA1 (*34*) (cc: 0.75; *P* < 0.001; fig. S3A) and neocortex (*20*) (cc: 0.46; *P* < 0.001; fig. S3B). In contrast, single-pair STG did not consistently correlate with the mean STG of non-peer pairs in neither the Before epoch (cc: 0.01; *P* = 0.902) nor the After epoch (cc: −0.05; *P* = 0.080; fig. S3, C and D), indicating that CA STG similarities extend beyond brain state changes. Furthermore, PYR-to-PYR firing synchrony was higher for same-CA pairs compared with different-CA pairs (*P* < 0.001, Wilcoxon’s paired test; fig. S4, A and B). Consequently, presynaptic PYRs within the same CA exhibit synchronous firing and similar STGs with the same postsynaptic PV cell.

To determine whether the STG change of a specific PYR-PV pair correlates with other same-CA changes, we compared for each pair the mean STG change of same-assembly peers (Fig. 2E). STG change of a single pair was correlated with the peer pairs STG change (cc: 0.49; *P* < 0.001, permutation test; Fig. 2F) but not with the non-peer STGs (cc: −0.02; *P* = 0.59; Fig. 2G). Similar results were observed in Control pairs that did not receive any light stimulation (fig. S3, E to H). Thus, PYR-PV pairs belonging to the same CA exhibit similar STG changes.

To determine whether the mean STG of individual CAs is balanced at the session level, we compared the mean STG change of each CA to the mean STG of simultaneously recorded CAs (Fig. 2H). The CA STG change did not consistently correlate with the mean STG change of other CAs (cc: −0.02; *P* = 0.58, permutation test; Fig. 2I). Moreover, STG changes of simultaneously recorded CAs were balanced and, similar to sham CAs, constructed by randomly shuffling the allocation of STGs to CAs (mean: −0.06, chance mean: −0.06, *P* = 0.53; SD: 0.36, chance SD: 0.46, *P* = 0.863; *n* = 27 sessions; Fig. 2J). However, inter-assembly variability of CAs (0.84) was higher than random CAs (SD, 0.46; *P* < 0.001, permutation test; Fig. 2K), indicating that STGs of same-CA pairs increase or decrease together. Thus, STGs of PYR-PV pairs that belong to the same CA change in a coordinated manner while preserving the equilibrium of the STG changes over multiple assemblies.

To quantify the predictive power of the assembly structure to STG changes, we used cross-validated support vector regression (SVR; Fig. 2L). In one SVR, we used the mean STG change of the peers and non-peers as an input (Fig. 2M, left). For the second SVR, we used randomly shuffled pairs from the same session (Fig. 2M, right). The reconstruction-based *R*^2^ of the individual PYR-PV STG change yielded by the first SVR was 0.48, consistently higher than the *R*^2^ yielded by the shuffled SVR (0.13; *P* < 0.001, *U* test; Fig. 2N).

A priori, excitability changes (*29*–*31*) may explain the observed simultaneous changes in STG within CAs. When the PV postsynaptic cell is more depolarized and exhibits a higher firing rate, all converging presynaptic PYRs will be more effective in triggering a spike (*34*). We used the changes in postsynaptic firing rates from the Before to the After epochs as a metric to quantify the changes in the excitability of the postsynaptic cells. Alterations in the postsynaptic cell firing rate from the Before epoch to the After epoch were correlated with STG changes (cc: 0.535; *P* < 0.001; permutation test; fig. S4F). To evaluate the individual contributions of peer pairs, non-peer pairs, and postsynaptic firing rate changes to predicting STG changes of a specific PYR-PV pair, we trained three separate SVRs while removing one feature at a time. *R*^2^s produced by all three SVRs were lower compared with the full model (trained using all three features; *P* < 0.001, Wilcoxon’s paired test; Fig. 2O). The lowest *R*^2^ was observed when peer pair information was removed. Thus, above and beyond the information carried by the changes in postsynaptic excitability, the STG changes of a single PYR-PV pair can be predicted by considering the assembly structure.

### Spike timing of the entire converging assembly predicts changes in spike transmission gain

**Fig. 3.**
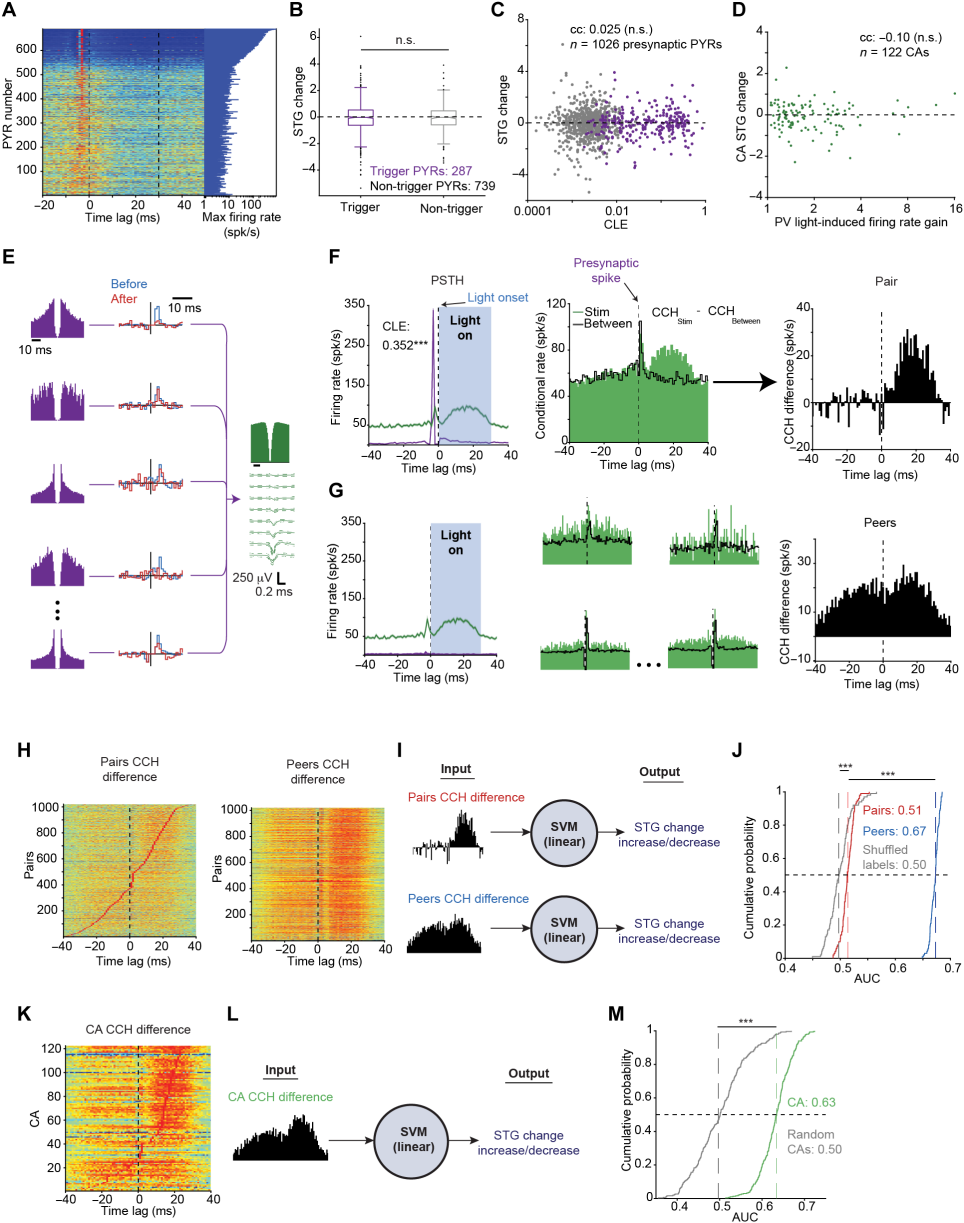
Spike timing of the entire converging assembly predicts changes in spike transmission gain. (**A**) PSTHs of all PYRs. (**B**) STG changes grouped by the presynaptic PYR (trigger/non-trigger). n.s., *P* > 0.05, *U* test. (**C**) STG changes versus CLE. Here and in (D), n.s., *P* > 0.05, permutation test. (**D**) Mean CA STG change versus PV light-induced gain. (**E**) Example CA with 11 PYR-PV pairs, 5 are shown. (**F**) Quantification of short-term spike timing changes. Left: PSTHs of the top PYR-PV pair, in which the PYR triggered closed-loop (CL) PV illumination. ***P < 0.001, Poisson test. Center: CCHs for the PYR-PV pair during illumination (“Stim”) and in the lack thereof (“Between”). Right: The CCH difference quantifies the effect on the two spike trains. (**G**) Left: PSTHs of four other PYRs and the PV cell. Center: CCHs. Right: Summing all peer CCH differences yields the peer’s CCH difference. (**H**) Pairwise and peer CCH differences. (**I**) Cross-validated binary classifier trained to predict the increase/decrease of pairwise STG. (**J**) Areas under the curve (AUCs) of 100 independently generated classifiers. Here and in (M), ***P < 0.001, *U* test. (**K**) CA CCH differences. (**L**) CA classification. (**M**) AUCs of the CA classifiers.

To determine whether spike timing influences the changes in STG within CAs, we examined the impact of immediate spike timing alterations during light stimulation on STG changes. During stimulation, firing rates of postsynaptic cells increased, exhibiting a median [IQR] light-induced firing rate gain of 1.56 [1.29 2.55] (*n* = 122; fig. S2E). Of all PYRs, [198 of 689 (29%)] were “trigger” PYRs, exhibiting a consistent peristimulus time histograms (PSTH) peak 3 [3, 3] ms before light onset (Fig. 3A), with a closed-loop efficiency (CLE) of 0.054 [0.013 0.184] (fig. S1, C and D). However, consistent PSTH peaks were not associated with higher STG changes (Fig. 3B), and the CLE of the PYRs did not consistently correlate with individual STG changes (cc: 0.02; *P* = 0.42; permutation test; Fig. 3C). Furthermore, postsynaptic firing-rate gain did not correlate with the mean CA STG changes (cc: −0.10; *P* = 0.69; Fig. 3D). Thus, the responses of the individual cells to light stimulation do not demonstrate a consistent correlation with the STG changes.

We explored whether the interaction of presynaptic and postsynaptic spike timing during light stimulation could predict STG changes. In a representative CA, 11 presynaptic PYRs converged on the same postsynaptic cell (Fig. 3E). Each pair may have experienced different spike patterns, affecting STG changes in distinct manners. Because of the correlation of STG changes within a CA (Fig. 2), we investigated how single-pair STG is affected by the spike timing of itself and the peers. To quantify the light-induced changes in spike patterns of one pair, we computed the conditional rate cross-correlation histogram (CCH) of a single PYR-PV pair during light stimuli (Fig. 3F). To quantify ongoing patterns, we computed the CCH for the same pair using spikes that occurred between stimuli (without illumination) during the Experience epoch. The difference between the CCHs during and between stimuli yields the “CCH difference” (Fig. 3F), capturing the specific effect of stimuli on spike timing during the Experience epoch. To quantify the effect of the stimuli on the spike timing of the peer pairs, we repeated the process for all other pairs in the same CA and summed all individual CCH differences (Fig. 3G).

For each pair participating in a CA with at least two presynaptic PYRs, we computed both the pair CCH difference and the peer CCH difference (1020 PYR-PV pairs; Fig. 3H). Consequently, the changes in spike timing resulting from light stimulation can be captured for a single pair and its peers using the CCH difference. To quantify the effect of light-induced spike timing changes on the single-pair STG changes, we trained cross-validated classifiers (support vector machines, SVMs) to predict whether single-pair STG change increases or decreases (Fig. 3I). The first classifier used the single-pair spike timing changes (pair-wise CCH differences; *n* = 1020 pairs), and the second classifier used the peer pairs spike timing changes (peers CCH differences). The classifier that used peer pairs CCH differences yielded a higher area under the curve (AUC), 0.673 [0.664 0.677], compared with the pairs classifier (AUC: 0.513 [0.506 0.521]; *P* < 0.001, *U* test; Fig. 3J). Similar results were observed when removing all assembly-based information (fig. S6). Thus, light-induced spike patterns of peers provide more information than the single-pair spike times regarding its own STG change.

To determine whether light-induced spike patterns can predict STG changes at the assembly level, we computed an assembly CCH difference for every CA by summing CCH differences over all pairs in the same assembly (*n* = 122 CAs; Fig. 3K). Classifiers trained on the assembly CCH differences (Fig. 3L) yielded an AUC of 0.634 [0.610 0.658], higher than the AUC yielded by the random CAs (0.497 [0.456 0.540]; *P* < 0.001, *U* test; Fig. 3M). Thus, changes in the spike timing of the entire CA predict STG changes at the assembly level.

### Precise timing carries more information than the initial conditions about changes in spike transmission

**Fig. 4.**
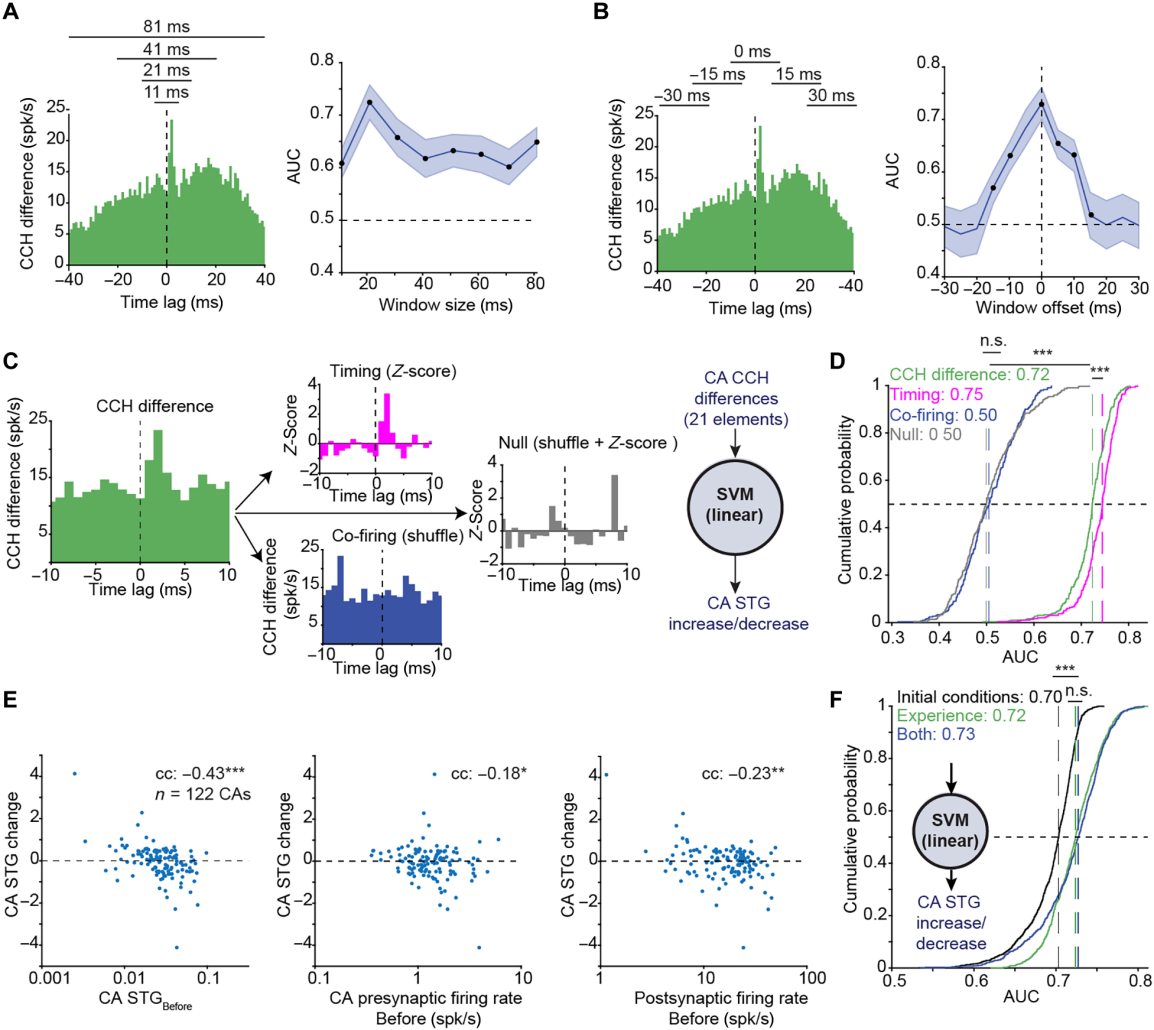
Precise timing carries more information than the initial conditions about changes in spike transmission. (**A**) Left: Example CA CCH difference. Right: Mean (solid line) and SD (band) of AUCs yielded by linear SVMs using different window sizes (*n* = 100 repetitions). All windows are centered at zero time lag. Here and in (B), black points indicate *P* < 0.05, Wilcoxon’s test corrected for multiple comparisons. (**B**) Same as (A), for different offsets of a 21-ms sliding window. (**C**) Disambiguating the contribution of precise spike timing and co-firing to the prediction of long-term STG changes. (**D**) AUCs for four classifiers based on the same 122 CA CCH difference vectors manipulated as in (C). Here and in (F), n.s., *P* > 0.05; ***P < 0.001, *U* test. (**E**) CAs STG change versus three initial conditions derived from the Before epoch. *P < 0.05; **P < 0.01; ***P < 0.001, permutation test. (**F**) AUCs produced by three classifiers predicting CA STG increase/decrease. Black, AUCs of a classifier that received as input the CA STG_Before_ and the CA PYR firing rate features as in (E). Green, AUCs yielded by a classifier that received only light-induced Experience epoch features, namely, the CA CCH differences at the optimized window of ±10 ms as in (D).

To determine the temporal resolution that affects the CA STG changes, we first narrowed down the temporal precision of the predictive spike patterns by using different segments of the CCH differences for classification. Expanding previous work in PYR-interneuron pairs without light-induced spike patterns (*35*), we found that the most accurate prediction of CA STG changes was obtained when a window of ±10 ms was centered at zero lag, yielding median [IQR] AUCs of 0.725 [0.706 0.751] (Fig. 4, A and B). Next, we compared the contribution of co-firing (at the timescale of the ±10-ms window) and millisecond-timescale spike timing by manipulating the CCH difference vectors. To remove all co-firing (“rate”) information, we *Z*-scored every vector (Fig. 4C, magenta). To remove all information about precise timing without modifying co-firing information, we shuffled the order of the 1-ms bins in the CCH difference vector (Fig. 4C, blue). When only co-firing information was maintained, classification was at chance level (AUC: 0.506 [0.455 0.546]; *P* = 0.85, Wilcoxon’s test; Fig. 4D, blue). However, when only timing information was maintained, classification yielded AUCs of 0.746 [0.718 0.763] (*P* < 0.001; Fig. 4D, magenta). Thus, millisecond-timescale light-induced changes of spike timing within PYR-PV CAs provide information about long-term STG changes.

If there is a connection between presynaptic and postsynaptic cells, then the initial connectivity strength may constrain further changes (*9*, *36*). Pairwise STG changes were negatively correlated with STG_Before_ (cc: −0.35; *n* = 1026 PYR-PV pairs; *P* < 0.001, permutation test; fig. S7A). Beyond the STG itself, other initial conditions (ICs) may constrain STG changes. Previous work in intact animals quantified CCH changes between presynaptic cells and postsynaptic interneurons as a function of firing rate changes (*19*). We found that STG changes were not consistently correlated with presynaptic firing rates during the Before epoch (cc: −0.05; *P* = 0.143; fig. S7B) but were negatively correlated with the initial postsynaptic firing rates (cc: −0.22; *P* < 0.001; fig. S7C). In sum, STG changes depend on the ICs.

To directly assess the relative contribution of the ICs and the light-induced spike patterns, we trained classifiers to predict the increase/decrease of the CA STG changes. As input, we used STG_Before_ and presynaptic firing rate (Fig. 4E) which optimized the IC-based predictions (fig. S7E), and the 21-element CA CCH differences which optimized the light-induced pattern predictions (Fig. 4, A and B). The classifier that used CA CCH differences alone yielded an AUC of 0.725 [0.706 0.751], higher than the classifier that used IC information alone (0.703 [0.682 0.717]; *n* = 122 CAs; *P* < 0.001, Wilcoxon’s test; Fig. 4F). Thus, light-induced precise spike patterns during the Experience epoch carry more information about assembly connectivity changes compared with the IC.

### Compared with excitability changes, spike timing contains additional information about transmission changes

We found that the spike timing of the presynaptic and postsynaptic cells in a CA during the experience epoch predicts the STG changes (Figs. 3 and 4). These findings were based on the CCH difference as a metric to capture short-term changes in spike timing during the Experience epoch. CCH differences allow disambiguating firing patterns during the light stimulation times from the intervening periods (Fig. 3F). Any slow changes of excitability should be common to both periods, resulting in flat CCH differences and no contribution to the prediction of STG changes. However, excitability changes may also contribute (Fig. 2O), and the relative contribution of spike timing and excitability changes is unknown.

**Fig. 5.**
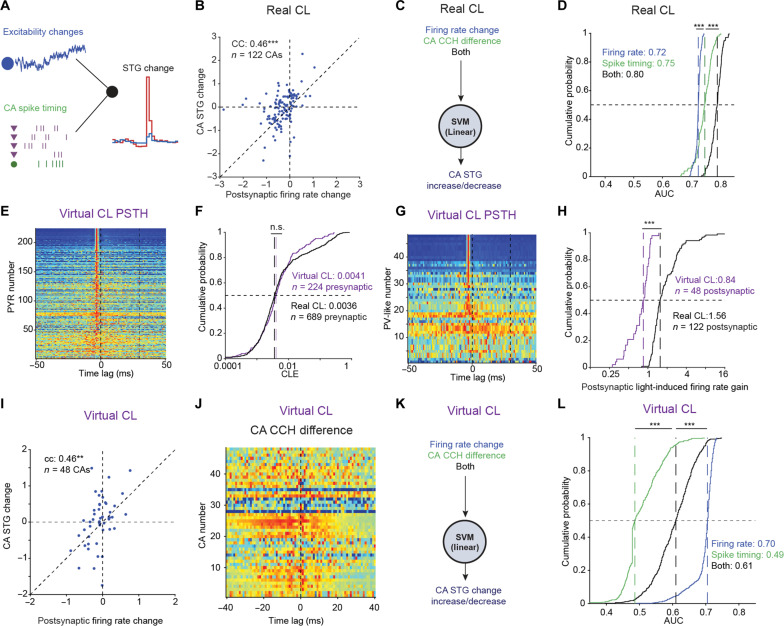
Compared with excitability changes, spike timing contains additional information about transmission changes. (**A**) Postsynaptic excitability and spike timing of the whole CA may affect STG changes. (**B**) CA mean STG changes versus postsynaptic firing rate changes for the real CL dataset. Postsynaptic firing-rate change is defined as the base-2 logarithm of the ratio between the firing rate during the After and Experience epochs. Here and in (I), ***P < 0.001, permutation test. (**C**) Three classifiers for predicting CA STG increase/decrease. (**D**) AUCs produced by classifiers as in (C). Here and in (F), (H), and (L), n.s., *P* > 0.05; ***P < 0.001, *U* test. (**E**) Virtual CL PSTHs of all 224 presynaptic cells in the Control dataset (table S3). Zero time lag represents the onset of the virtual CL stimulus. (**F**) CLEs for all virtual CL presynaptic [derived from the PSTHs in (E)] and real CL presynaptic cells (as in fig. S1D). (**G**) PSTHs of all 48 postsynaptic cells in the Control dataset. (**H**) Distribution of light-induced firing rate gain for the virtual CL postsynaptic cells [derived from the PSTHs in (G)] and for the real CL postsynaptic cells (as in fig. S2E). (**I**) CA mean STG changes versus postsynaptic firing rate changes for the virtual CL dataset. (**J**) Scaled CA CCH differences for the 48 CAs in the virtual CL dataset. (**K** and **L**) Classifiers and AUCs for the virtual CL dataset.

First, we assessed the relative contribution of spike timing and gradual excitability changes of the postsynaptic cell during the Experience period. We quantified postsynaptic excitability changes by the base-2 logarithm of the ratio between the firing rates during the After and Experience epochs. Alterations in postsynaptic firing rates were correlated with the CA mean STG changes (cc: 0.46; *P* < 0.001; permutation test; Fig. 5B), suggesting that excitability changes provide a major contribution to the STG changes. We then directly compared the predictive power of the postsynaptic firing rate changes with the predictive power of the light-induced spike patterns using three different classifiers. As input, the first classifier used postsynaptic firing-rate changes, the second classifier used the *Z*-score optimized window of ±10-ms CCH differences (as in Fig. 4D, blue curve), and the third classifier used both features (Fig. 5C). We found that the classifier trained on the postsynaptic firing-rate changes yielded AUCs of 0.725 [0.721 0.730] (Fig. 5D, blue curve), lower than the classifier trained solely on spike timing information (AUC: 0.746 [0.718 0.763]; Fig. 5D, green curve; *P* < 0.001, *U* test). The classifier using both spike timing and postsynaptic firing rate change information yielded AUCs of 0.796 [0.776 0.803] (Fig. 5D, black curve), consistently higher than the other two classifiers (*P* < 0.001, *U* test). Similar results were obtained for alternative definitions of excitability changes (fig. S8, A and B). Thus, spike timing as quantified by the CCH differences provides additional information and a more accurate prediction of the future STG changes, compared with the concurrent changes of postsynaptic cell excitability.

Second, we assessed the possible leakage of information from gradual excitability changes of the postsynaptic cell to the CCH differences during the Experience epoch. For that purpose, we tested whether spike timing during the Experience epoch can be used to predict the mean STG changes of the assembly in the Control dataset, in which no closed-loop stimulation was applied. If slow excitability changes somehow leak into the CCH difference analysis, then CCH differences derived from randomly selected periods in the Control dataset would provide above-chance predictions of the STG changes. To test the possibility, we computed CA CCH differences during the Control sessions. We conducted “virtual closed-loop” experiments, randomly selecting a trigger presynaptic PYR and stimulation periods according to the empirical distribution of CLEs observed during Stimulation (real closed-loop) sessions (Fig. 5, E to H). As in the real closed-loop dataset (Fig. 5B), postsynaptic firing rate changes were correlated with the STG changes in the virtual closed-loop experiments (cc: 0.46; *P* < 0.001; permutation test; Fig. 5I). Next, we repeated the analysis of Fig. 5D using the CCH differences and postsynaptic firing rate changes derived from the virtual closed-loop dataset (Fig. 5, J and K). The classifier trained on the postsynaptic excitability change feature yielded AUCs of 0.705 [0.693 0.714] (Fig. 5L, blue curve). However, the classifier which used CCH differences yielded AUCs of 0.486 [0.479 0.542] (*P* < 0.001, *U* test; Fig. 5L, green curve), indicating that spike timing does not predict STG changes in the Control dataset. Similar results were obtained for alternative definitions of excitability changes (fig. S8, C and D). Thus, CCH differences are not susceptible to ongoing changes in excitability and cannot predict the observed changes in STG in the Control dataset.

## DISCUSSION

We found that precise spatiotemporal spike patterns generated by closed-loop optogenetic manipulations lead to long-lasting modifications of spike transmission among PYR-PV pairs. Spike transmission changes occur concurrently across multiple PYR-PV pairs within the same CA. Changes in STGs are more accurately predicted by spike patterns that include the same-assembly peers, compared with the specific pair. Modifications of STGs are constrained by the initial spike transmission and presynaptic firing rate, and influenced by postsynaptic excitability changes. However, the impact of the ICs is smaller than the effect of spike timing, which makes an independent contribution.

### Plasticity in converging assemblies

We focused on the plasticity of spike transmission between PYRs and PV cells in CAs recorded in hippocampal region CA1 (*18*, *20*). Previous research demonstrated that PYR-interneuron networks in CA1 play a crucial role in the emergence and reorganization of place fields (*21*, *37*) and memory regulation (*38*). Furthermore, experience-dependent plasticity has been observed in neocortical (*39*, *40*) and hippocampal PYR-interneuron networks (*40*–*42*). We found that STGs in the same CA are similar to begin with, i.e., “wired together.” The very organization of cells into assemblies based on postsynaptic neurons is also influenced by anatomical (*43*), functional (*44*), and embryological (*45*) properties. Furthermore, we found that same-assembly connections undergo simultaneous same-direction modifications, i.e., “change together.” These findings are consistent with theories suggesting that neural coding is facilitated by cell assemblies linked together by dynamic changes in synaptic weights (*46*–*50*). Our results highlight the role of CAs as natural building blocks of neuronal codes, facilitated by simultaneous changes in connectivity of CAs.

A closed-loop approach was used to pair a single spike of a presynaptic neuron with the spiking activity of the postsynaptic neuron (*32*). The approach differs from previous in vivo STDP experiments, which used sensory stimuli to pair the activity of a postsynaptic cell with the activity of a population of presynaptic and other cells (*13*–*15*, *17*, *51*). The present approach allows examining STG changes for each pair while taking into account other connections as well. The observation that STG changes occur concurrently for all converging connections may not be exclusive to PYR-PV CAs (*30*). The same mechanism may underlie the plasticity reported in other in vivo STDP studies, where the spike timing of the entire CA influences all of the constituent connections.

### Underlying mechanisms

The concurrent changes of spike transmission among a PYR-to-PV CA are consistent with prior slice work in PYR-to-basket cells in the CA1 pyramidal cell layer (*23*, *24*) and in other excitatory connections onto inhibitory cells (*36*). Mechanisms underlying heterosynaptic plasticity include excitability-based processes and calcium-based processes. Excitability-based mechanisms may be cellular (*30*) or compartmental (*29*, *52*). Likewise, changes in intracellular calcium may be localized to micro-regions (*53*–*55*) or be more widespread (*26*, *27*). Thus, while molecular-cellular infrastructure for both concurrent and counter-balancing heterosynaptic changes exists, the present findings show that in converging PYR-to-PV assemblies of the intact brain, the concurrent changes dominate.

Mechanisms involving postsynaptic excitability are likely to contribute to the concurrent STG changes (*29*–*31*). We demonstrated that changes in postsynaptic excitability, quantified by changes in postsynaptic firing rate, predict STG changes (Figs. 2O and 5). While we found that nonspecific excitability changes are a major contributor to STG changes, our results also show that assembly spike timing predicts STG modifications independently of any ongoing excitability changes (Fig. 5D). Thus, firing rate changes alone could not explain the present findings (Figs. 3D and 5 and figs. S7 and S8).

Consistent with the present extracellular observations, nearly all prior studies that buffered intracellular calcium indicated necessity (*24*, *56*–*59*). In interneurons, the source of calcium transients can be intracellular stores or extracellular, mediated by *N*-methyl-d-aspartate (NMDA) receptors, calcium-permeable AMPA receptors, or group I metabotropic glutamatergic receptors (mGluR). In vitro, mGluR have been involved in the plasticity of excitatory connections onto dentate gyrus fast-spiking neurons (*58*), oriens lacunosum/moleculare interneurons (*60*), and neocortical fast-spiking neurons (*61*), but not CA1 str. pyramidale PV cells. While blockade of NMDA receptors does not prevent plasticity in CA1 str. pyramidale PV cells in vitro, calcium-permeable AMPA receptors are required (*57*, *62*). On the basis of these observations, we hypothesize that intracellular calcium changes mediated by calcium-permeable AMPA receptors mediate the concurrent changes of spike transmission in CAs.

### Timescale of plasticity

The induced short-term spike patterns between PYR-PV pairs had a long-lasting impact on the STG, highlighting the role of spike timing in synaptic plasticity. The patterns that predicted the changes occurred on a timescale of milliseconds, consistent with the effects of spike timing observed in STDP studies in vitro (*9*–*11*, *36*, *63*) and in vivo (*14*, *15*, *17*, *64*). Modifying synaptic connections based on experienced spike timing at short timescales has many theoretical and practical advantages (*6*, *8*, *65*–*67*).

However, highly precise patterns at short timescales leave open the question of how associations are established over behaviorally relevant timescales spanning seconds (*68*). Recent work proposed a form of plasticity that operates on a longer timescale (*16*, *22*, *69*), “behavioral timescale synaptic plasticity” (BTSP). In BTSP, seconds-long plateau potentials of a single hippocampal PYR trigger the formation of place fields by altering connectivity with innervating CA3 input (*16*, *70*). The apparent discrepancy between the millisecond-timescale CA findings and the second-timescale BTSP results may be attributed to different intrinsic properties of PYR-PYR and PYR-PV CAs. A second manner to settle the apparent timescale discrepancy is to consider that in the intact hippocampus, long plateau potentials necessarily overlap other ongoing events. For instance, during theta oscillations, spikes of presynaptic PYRs and postsynaptic interneurons are organized in precise sequences at millisecond timescale (*71*–*75*). The timescale of multi-neuronal spiking during BTSP is presently unknown since spikes of CA3 and CA1 PYRs were not recorded simultaneously. We hypothesize that BTSP requires short-timescale spiking patterns of presynaptic CA3 and postsynaptic CA1 PYRs. The hypothesis may be tested by recording PYR spiking in CA3 and CA1 during the induction of CA1 place fields, potentially elucidating the timescale of spiking activity during the behaviorally-relevant alternation of synaptic connections.

## MATERIALS AND METHODS

### Experimental subjects

A total of seven freely moving male mice were used in this study (table S1). The mice were aged 16 [8,30] weeks (median, [range]) at the time of implantation. Animals were healthy, were not involved in previous procedures, and weighed 28.6 [22.7,30.1] g at the time of implantation. Four mice expressed ChR2 in PV cells under the PV promoter (PV::ChR2), achieved by crossing driver PV-Cre males (JAX #008069, The Jackson Laboratory) with ChR2 reporter females (Ai32; JAX #012569). Only these mice were used for optogenetic stimulation. Two mice expressed ChR2 in PYRs, generated by crossing CaMKII-Cre males (JAX #005359) with Ai32 females (CaMKII::ChR2). One mouse expressed ChR2 in somatostatin cells, generated by crossing an SST-Cre male (JAX #013044) with an Ai32 female (SST::ChR2). Mice were single-housed to prevent damage to the implanted apparatus. All animal handling procedures were in accordance with Directive 2010/63/EU of the European Parliament, complied with Israeli Animal Welfare Law (1994), and approved by Tel Aviv University Institutional Animal Care and Use Committee (IACUC #01-16-051 and #01-20-049).

### Probes and surgery

Every animal was implanted with a multi-shank silicon probe attached to a movable microdrive and coupled with optical fibers for optogenetic stimulation as previously described (*32*). Briefly, light was delivered to every optical fiber by butt-coupling a 2-mm-diameter light-emitting diode (LED) to the fiber. Every fiber was then glued to one of the probe shanks. Every probe was equipped with four diode-coupled fibers. The current driving each LED was generated by a linear current source, controlled by an analog voltage signal generated by a real-time processor (RX8, Tucker-Davis Technologies) as previously described (*32*). When driven by a 50-mA current, light power measured at the tip of the fiber was 42 ± 11 μW (means ± SD over *n* = 16 LEDs).

The probes used were Buzsaki32 (NeuroNexus), Stark64 (Diagnostic Biochips), Dual-sided128 (Diagnostic Biochips), Dual-sided64 (Diagnostic Biochips), and Stark128 (Diagnostic Biochips). The Buzsaki32 probe consists of four 52-μm-wide, 15-μm-thick shanks, spaced horizontally 200 μm apart, with each shank consisting of eight recording sites, spaced vertically 20 μm apart. The Stark64 probe consists of six 48-μm-wide, 15-μm-thick shanks, spaced horizontally 200 μm apart, with each shank consisting of 10 to 11 recording sites, spaced vertically 15 μm apart. The Dual-sided128 probe consists of two Stark64 probes attached back-to-back, yielding six 48-μm-wide, 30-μm-thick dual-sided shanks. The Dual-sided64 probe consists of two 70-μm-wide, 30-μm-thick dual-sided shanks, spaced horizontally 250 μm apart, with every side of each shank consisting of 16 channels spaced vertically 20 μm apart. The Stark128 probe consists of eight 33.5-μm-wide, 15-μm-thick shanks, spaced horizontally 125 μm apart, with each shank consisting of 16 recording sites, spaced vertically 15 μm apart. All probes were implanted in the neocortex above the right hippocampus (PA/LM, −1.6/1.1 mm; 45° angle to the midline) under isoflurane (1%) anesthesia as previously described (*76*). After every recording session, the probe was translated vertically downwards by up to 70 μm. Analyses included only recordings from the CA1 pyramidal cell layer, recognized by the appearance of multiple high-amplitude units and spontaneous isopotential ripple events.

### Recording sessions

Neuronal activity was recorded in 4.3 [3.8 4.8] hour sessions (median [IQR]). Animals were equipped with a three-axis accelerometer (ADXL-335, Analog Devices) for monitoring head movements. Head position was tracked in real time using two head-mounted LEDs, a machine vision camera (ace 1300-1200uc, Basler), and a dedicated system (*77*). Every session started with a baseline neural recording of at least 15 min, while the animal was in the home cage or in a 0.8-m-diameter open field. Following the baseline recordings, a Before epoch that lasted a median [range] of 45 [40,45] min was carried out, during which the animals were free to behave but no light stimuli were applied. Following the Before epoch, the Experience epoch began, which lasted 55 [29,80] min. During the Experience epoch, the Stimulation group consisting of four PV::ChR2 mice (*n* = 31 sessions in four mice; table S2) received closed-loop optogenetic stimuli, whereas the Control group (*n* = 11 sessions in six mice; table S3) underwent continuous recording without any stimuli. All the optogenetic manipulations were carried out during the Experience epochs of the 31 sessions recorded from the four PV::ChR2 mice (table S2). The Control dataset (table S3) was derived from different sessions and did not include any light stimuli. Following the Experience epoch, the After epoch was carried out without any stimuli, lasting 45 [40,45] min.

### Closed-loop stimulation

For the execution of closed-loop stimulation, we selected a PYR spike waveform to serve as a trigger. To select the trigger PYR, we manually picked up to four same-shank channels, used for detecting and acquiring spikes over a duration of 2 to 3 min with a real-time digital signal processor operating at 24414 Hz (RX8, Tucker-Davis Technologies). After a batch of spikes was collected, spikes were sorted offline by applying principal components analysis (PCA) to each channel, followed by the use of the K-means algorithm on the three first PCA coefficients extracted from each channel to form clusters. We then selected a spike cluster for parameter extraction during real-time detection. We manually selected three voltage windows, using the same channels used for real-time spike detection. Each window consisted of two voltage points at one time point. After uploading the definitions to the digital signal processor, only spikes that passed through all the windows were detected. Upon the real-time detection of a PYR spike, a voltage command was issued to a linear current source, prompting the delivery of a 30-ms light stimulus. In 6 out of the 29 Stimulation sessions (one to two sessions in every mouse), we chose a single PYR for closed-loop illumination. In the rest of the sessions, we ran two closed-loop experiments in parallel. Because the selection was done online and used partial information (only four same-shank channels), the triggering efficiency was never perfect and the actual number of PYRs that were involved in triggering the illumination was always larger than one. Offline, we quantified the fraction of spikes of every unit used to generate light stimuli using the CLE (see below; fig. S1B). We then assigned a *P* value to every PYR in every session. The median [IQR] CLE was 0.003 [0.001 0.008] (*n* = 1853 PYRs; fig. S1C). The number of “trigger PYRs” (i.e., PYRs with a CLE *P* < 0.001; fig. S1D) was 11 [5 18] (*n* = 29 sessions; fig. S1E). Further post hoc analysis indicated that light stimuli were given a median [IQR] of 3 [3 3] ms (*n* = 394,886 light stimuli) after the spike trough occurred. Following a light stimulus, a dead time of 20 ms was applied during which no stimulation was given.

### Spike detection, spike sorting, and cell type classification

Neural activity was filtered, amplified, multiplexed, digitized on the headstage (0.1 to 7500 Hz, ×192; 16 bits, 20 kHz; RHD2132 or RHD2164, Intan Technologies), and recorded by an RHD2000 evaluation board (Intan Technologies). Offline, spikes were detected and sorted into single units automatically using either KlustaKwik3 (*78*) or KiloSort2 (*79*). Automatic spike sorting was followed by manual adjustment of the clusters. Only well-isolated units were used for further analyses [amplitude, >40 μV; L-ratio, <0.05; inter-spike interval index, <0.2; (*20*)]. Units were then classified into putative PYR or PV-like interneurons using a Gaussian mixture model (*33*).

### Selection of a subset of data

To select PYR-PV pairs for the Stimulation and the Control groups, we applied the following criteria: (i) Detection of an excitatory monosynaptic connection in the CCH using spike trains from the combined Before and After epochs (*P* < 0.001, Poisson test). (ii) Accumulation of at least 400 counts in the count CCHs of each of the Before and After epochs at the −30 < τ ≤ 30 range. (ii) Identification of the excitatory monosynaptic peak (0 < τ ≤ 5 range) as the highest peak in the Before or After CCH. The Stimulation group included only pairs in which the postsynaptic cell was a PV cell (i.e., activated by the closed-loop stimulation).

The Stimulation group consisted of 1838 PYRs and 420 interneurons recorded during 31 sessions from four mice, forming 24,461 pairs. Of these, 3702 (15%) PYR-interneurons pairs were connected (*P* < 0.001, Poisson test). After applying the above criteria, the group consisted of 689 PYRs, 122 PV cells, and 1026 connected pairs from 29 sessions in the four mice. The Control group consisted of 547 PYRs and 152 interneurons recorded during 11 sessions from six mice, forming 10,743 pairs. Of these, 1197 (11%) pairs were connected. After applying the criteria, the group consisted of 224 PYRs, 48 interneurons, and 388 connected pairs from nine sessions in five mice.

### Quantification of closed-loop feedback: Closed-loop efficiency and light response

To determine what fraction of spikes of a given unit were used to generate light stimuli, we defined a “closed-loop efficiency” (CLE) measure. To estimate the CLE, we used the same approach as for computing the STG (see below), based on the PSTHs (1-ms bin size) instead of the CCH. The PSTHs were constructed around stimulus onset for every PYR in the Stimulation group and scaled to spikes per second (spk/s; as in Fig. 3A). The CLE was calculated as the area under the peak in the −5 ≤ τ < 0 ms region of interest (ROI), and the baseline activity was determined by hollowed median filtering (5-ms half-width) of the PSTH. The CLE is limited to the [0,1] range. A CLE of zero indicates that no spikes were followed by light stimuli, and a CLE of 1 indicates that every spike was followed by a single light stimulus. In practice, the maximal CLE is lower than 1 even for perfect detection due to the processing delay (3 ms), stimulus duration (30 ms), and post-stimulus dead-time (20 ms). The CLEs were 0.004 [0,0.848] (median [range]; *n* = 689 PYRs). In trigger PYRs, defined as PYRs with a consistent peak in the −5 ≤ τ < 0 ms ROI of the PSTH (*P* < 0.001, Poisson test), the CLEs were 0.054 [0.002,0.848] (*n* = 198; fig. S2C).

To identify PV cells that were light-activated by the closed-loop stimulation, we constructed PSTHs (bin size of 1 ms) around the stimulus onset for every PV cell (as in Fig. 1J). PV cells that exhibited a consistent increase in firing rate during the 10 ≤ τ < 30 ms interval relative to baseline activity (*P* < 0.05, Poisson test) were classified as light-activated PV cells. The baseline activity was determined by the mean firing rate during a 15-ms period starting 30 ms before light onset and ending 15 ms before light onset.

### Computing STG changes

To compute STG changes, two count CCHs (0.5-ms bins) were constructed for every pair, separately for the Before and After epochs. The STG was then computed for each of the two CCHs as previously described (*34*). Briefly, the spike transmission curve was estimated by the difference between the deconvolved CCH and the baseline, determined by hollowed median filtering of the count CCH, scaled to spk/s. The STG was defined as the area under the peak in the monosynaptic temporal ROI (0 < τ ≤ 5 ms), extended until the causal zero-crossing points. The STG change was then computed as the base-2 logarithm of the ratio between the STG_After_ and the STG_Before_. The STG change is not limited to a specific range. An STG change of 1 (or −1) indicates that the STG has doubled (or halved) following the Experience epoch, while a value of 0 indicates no change in STG.

To assess the consistency of changes in STG (i.e., a significant increase or decrease), we conducted a permutation test. First, we generated a binary spike time lag matrix (0.5-ms bins) for each PYR-PV pair using spikes that occurred during the Before and After epochs. Each row in the matrix represented a single PYR spike, and every column denoted a time lag. The value in every cell indicated whether a PV spike occurred (1) or not (0) during the corresponding time interval. Every row was labeled according to the source spike (Before or After). The original STG change was computed from the count CCHs generated by summing the rows that correspond to the Before epoch and the After epoch separately. Second, we shuffled the row labels of the matrix and partitioned the matrix into two matrices with identical sizes to the original matrices, but according to the shuffled labels. We then computed the STG change using the two shuffled matrices. Third, we repeated the shuffling process 2000 times and obtained a distribution of STG changes, which was used to estimate the two-tailed *P* value of the original (observed) STG change. Specifically, the *P* value was the fraction of shuffled STG changes that were more extreme than the observed STG change (i.e., either higher or lower). The fraction was calculated by adding 1 to the number of more extreme STG changes and dividing by the total number of shuffles plus 1.

We found that 11% (116 of 1026) of the Stimulation pairs exhibited a consistent STG increase (*P* < 0.001, binomial test comparing to chance level, 2.5%), and 16% (160 of 1026) exhibited a decrease (*P* < 0.001). Similar results were also observed in the Control pairs group, recorded during long no-stimulus durations. Of the Control pairs, 14% (54 of 388) exhibited a consistent STG increase (*P* < 0.001), and 12% (45 of 388) exhibited a decrease (*P* < 0.001).

### Synchrony quantification

To quantify synchrony between a pair of PYR spike trains s_1_ and s_2_, we constructed the count CCH using 0.5-ms bins. We then counted the number of coincident counts in the synchrony ROI (−1 ≤ τ ≤ 1 ms) *n*_sync_, and divided that by the geometric mean total number of spikes in each of the two trains, η_sync_ *= n*_sync_*/√N*_1_*N*_2_. The η_sync_ measure generalizes the synchrony effect size of (*80*) to the symmetric setting. η_sync_ is bounded 0 ≤ η_sync_ ≤ 1, approaching 1 when all spikes of one of the trains are synchronous with the other train. Notably, η_sync_ may be nonzero simply by chance, even when using a small bin size and a small ROI, and even when there is no millisecond-timescale synchrony. To obtain an estimate of the synchrony above expected by chance, we defined chance level using timescale separation, by hollowed median filtering (5-ms half-width) of the CCH, obtaining a predictor CCH, pred (*34*). We then derived the chance level synchrony effect size from the predictor in the same manner, η_pred_ *= n*_pred_*/√N*_1_*N*_2_. The synchrony measure Sync is then defined as Δη *=* η_sync_ − η_pred_, and is bounded −1 ≤ Δη ≤ 1. To determine significance, we used a Poisson test, estimating the Poisson probability of observing *n*_sync_ or more counts when *n*_pred_ are expected and applying a continuity correction (*80*).

We measured pairwise synchrony for every PYR in the set of *n* = 689 PYRs during the entire recording session (excluding stimulation times) using the abovementioned bin size and ROI. After excluding same-shank PYRs, the mean Sync was computed for the target PYR and all peers (PYRs that participated in the same CA) and for all non-peer PYRs. We also counted the fraction of synchronized peers and synchronized non-peers (*P* < 0.001, Poisson test). A total of 623 of 689 PYRs had at least one peer and one non-peer recorded on other shanks. The median [IQR] number of peers per PYR was 10 [6 19], and the median [IQR] mean Sync with peers was 0.0014 [0.0008 0.0025] (*n* = 623 PYRs). For non-peers, there were 16 [8 29] non-peers per PYR, and the mean Sync was lower at 0.0010 [0.0007 0.0016] (*P* < 0.001, Wilcoxon’s paired test; fig. S4A). The fraction of synchronized peers per PYR was 26% [8% 46%], higher than the fraction of synchronized non-peer pairs per PYR (16% [5% 36%]; (Wilcoxon’s paired test; fig. S4B). Considering all PYR-PYR pairs, the fraction of synchronized peer pairs was 31% (2398 of 7692 pairs), higher than the overall fraction of synchronized non-peer pairs (2239 of 11,211 pairs, 20%; *P* < 0.001, *G* test). The results were not sensitive to specific parameter values, and similar (and significant) results were obtained for bin sizes of {0.25, 0.5, 1} ms, and for ROIs of {−0.5 ≤ τ ≤ 0.5, −1 ≤ τ ≤ 1, −1.5 ≤ τ ≤ 1.5} ms.

We measured PV-PV synchrony and the changes thereof between the Before and After epochs in the Stimulation data. To quantify synchrony changes, we first quantified synchrony during the Before and After epochs for every pair of PV cells recorded simultaneously on distinct shanks (see example in fig. S5, A to C). The *n* = 122 CAs recorded during *n* = 29 sessions consisted of *n* = 237 pairs of PV cells recorded on distinct shanks, with a median of five pairs per session (fig. S5D, inset). Of these, 190 of 237 (80%) pairs exhibited significant synchrony during the Before epoch (*P* < 0.001, Poisson test), and 184 of 237 (78%) during the After epoch. We then plotted Sync_After_ versus Sync_Before_ (fig. S5D) and computed the “Sync change,” defined as the base-2 logarithm of the ratio between Sync_After_ and Sync_Before_ (fig. S5E). The median [IQR] Sync change was −0.028 [−0.33 0.35] (*P* = 0.99, Wilcoxon’s test comparing to a zero null), not consistently different from the median of the STG changes (median STG change: −0.028; *P* = 0.40, *U* test; fig. S5E, blue curve). However, the magnitude of the Sync changes was 0.335 [0.152 0.825], lower than the magnitude of the STG changes (0.556 [0.240 1.079]; *P* < 0.001, *U* test; fig. S5F). Thus, both PYR-to-PV transmission and PV-to-PV synchrony maintain equilibrium at the population level, and the magnitude of the changes is higher among PYR-PV pairs.

### Cross-validated regression

To predict a single STG change (Fig. 2, M to O), we used an SVR with a radial basis function kernel. The inputs for the SVR were different combinations of the following three features: (i) the mean STG changes of peer pairs, (ii) the mean STG changes in non-peer pairs, and (iii) changes in the firing rate of the postsynaptic cell. We conducted training and testing of the SVR using a fivefold cross-validation method. The entire process was reiterated 100 times, each instance partitioning the data into five random segments. The coefficient of determination (*R*^2^) for each fivefold cross-validation was computed to evaluate the performance under cross-validation. The chance SVR was used as an input of the mean STGs of pairs from the same session, the labels of which were randomly shuffled into peer pairs and non-peer pairs groups (Fig. 2, M and N). To assess the individual contribution of peers, non-peers, and the firing rate changes for predicting the single STG change, we repeated the process by training additional SVRs, each with only two of the features (Fig. 2O).

### Binary classification

To predict whether the STG change increased or decreased following the Experience (i.e., between the Before and After epochs), we used a linear SVM. To evaluate classification performance, we trained every classifier using fivefold cross-validation, repeating the process 100 times with the data split into five random folds each time. We then calculated the AUC of each fivefold cross-validation. AUC values were compared to the corresponding values yielded by a control SVM. The control SVM used shuffled labels (Fig. 3J and fig. S7D), shuffled CAs (Fig. 3M), *Z*-scored CCH differences with shuffled bins (Fig. 4D), or virtual closed-loop data from Control sessions (Fig. 5L).

Because STGs in a given CA change together (Fig. 2), the input used for the SVM classifier may contain information about the association of individual pairs to CAs, which could affect the prediction. To eliminate any assembly-based information from the classification process, we used two approaches: entire CA cross-validation or subsampling. In the first approach, entire CA cross-validation (fig. S6A), we divided the data into five folds of identical sizes, subject to the constraints of the data, with each fold containing data from the entire CAs. In other words, pairs from the same assembly were used either for training or for testing, but never for both. Thus, the training and testing were applied to data from different assemblies. In the second, subsampling approach (fig. S6B), we randomly selected one pair from each CA, resulting in 116 pairs. We then trained an SVM using many different partitions of random fivefold cross-validation with the 116 pairs. We repeated the process until every pair was sampled at least 100 times. As some pairs were sampled more than others, we randomly selected 100 predictions for each pair.

### Statistical analyses

In all statistical tests, a significance threshold of α = 0.05 was used. An exception was the threshold used for determining whether two units exhibit monosynaptic connectivity or synchrony (α = 0.001). In all cases, nonparametric testing was used. All statistical details (*n*, median, IQR, range, mean, and SD) can be found in the main text, figures, figure legends, and tables. To estimate whether fractions were larger or smaller than expected by chance, an exact binomial test was used (two-tailed). Differences in the proportions of two categorical variables were tested with a likelihood ratio test (*G* test). Differences between two group medians were tested with either the Mann-Whitney *U* test (unpaired samples) or Wilcoxon’s paired signed-rank test (two-tailed). To estimate whether a median was larger or smaller than expected by chance, Wilcoxon's signed-rank test was used (two-tailed). The association between parameters was quantified using Spearman’s rank correlation and tested with a permutation test. For all figures, n.s., *P* > 0.05; *P < 0.05; **P < 0.01; ***P < 0.001.

## References

[1] M. Pignatelli, G. K. E. Umanah, S. P. Ribeiro, R. Chen, S. S. Karuppagounder, H.-J. Yau, S. Eacker, V. L. Dawson, T. M. Dawson, A. Bonci, Synaptic plasticity onto dopamine neurons shapes fear learning. Neuron 93, 425–440 (2017). 28103482 10.1016/j.neuron.2016.12.030

[2] P. R. Roelfsema, A. Holtmaat, Control of synaptic plasticity in deep cortical networks. Nat. Rev. Neurosci. 19, 166–180 (2018). 29449713 10.1038/nrn.2018.6

[3] J. C. Magee, C. Grienberger, Synaptic plasticity forms and functions. Annu. Rev. Neurosci. 43, 95–117 (2020). 32075520 10.1146/annurev-neuro-090919-022842

[4] G. Neves, S. F. Cooke, T. V. P. Bliss, Synaptic plasticity, memory and the hippocampus: A neural network approach to causality. Nat. Rev. Neurosci. 9, 65–75 (2008). 18094707 10.1038/nrn2303

[5] N. Caporale, Y. Dan, Spike timing–dependent plasticity: A Hebbian learning rule. Annu. Rev. Neurosci. 31, 25–46 (2008). 18275283 10.1146/annurev.neuro.31.060407.125639

[6] D. E. Feldman, The spike-timing dependence of plasticity. Neuron 75, 556–571 (2012). 22920249 10.1016/j.neuron.2012.08.001 PMC3431193

[7] J. E. Lisman, Bursts as a unit of neural information: Making unreliable synapses reliable. Trends Neurosci. 20, 38–43 (1997). 9004418 10.1016/S0166-2236(96)10070-9

[8] S. Song, K. D. Miller, L. F. Abbott, Competitive Hebbian learning through spike-timing-dependent synaptic plasticity. Nat. Neurosci. 3, 919–926 (2000). 10966623 10.1038/78829

[9] G. Bi, M. Poo, Synaptic modifications in cultured hippocampal neurons: Dependence on spike timing, synaptic strength, and postsynaptic cell type. J. Neurosci. 18, 10464–10472 (1998). 9852584 10.1523/JNEUROSCI.18-24-10464.1998 PMC6793365

[10] R. C. Froemke, Y. Dan, Spike-timing-dependent synaptic modification induced by natural spike trains. Nature 416, 433–438 (2002). 11919633 10.1038/416433a

[11] J. C. Magee, D. Johnston, A synaptically controlled, associative signal for Hebbian plasticity in hippocampal neurons. Science 275, 209–213 (1997). 8985013 10.1126/science.275.5297.209

[12] K. K. L. Pang, M. Sharma, K. Krishna-K, T. Behnisch, S. Sajikumar, Long-term population spike-timing-dependent plasticity promotes synaptic tagging but not cross-tagging in rat hippocampal area CA1. Proc. Natl. Acad. Sci. U.S.A. 116, 5737–5746 (2019). 30819889 10.1073/pnas.1817643116 PMC6431168

[13] E. Ahissar, E. Vaadia, M. Ahissar, H. Bergman, A. Arieli, M. Abeles, Dependence of cortical plasticity on correlated activity of single neurons and on behavioral context. Science 257, 1412–1415 (1992). 1529342 10.1126/science.1529342

[14] Y.-X. Fu, K. Djupsund, H. Gao, B. Hayden, K. Shen, Y. Dan, Temporal specificity in the cortical plasticity of visual space representation. Science 296, 1999–2003 (2002). 12065829 10.1126/science.1070521

[15] V. Pawlak, D. S. Greenberg, H. Sprekeler, W. Gerstner, J. N. Kerr, Changing the responses of cortical neurons from sub- to suprathreshold using single spikes in vivo. eLife 2, e00012 (2013). 23359858 10.7554/eLife.00012 PMC3552422

[16] L. Z. Fan, D. K. Kim, J. H. Jennings, H. Tian, P. Y. Wang, C. Ramakrishnan, S. Randles, Y. Sun, E. Thadhani, Y. S. Kim, S. Quirin, L. Giocomo, A. E. Cohen, K. Deisseroth, All-optical physiology resolves a synaptic basis for behavioral timescale plasticity. Cell 186, 543–559.e19 (2023). 36669484 10.1016/j.cell.2022.12.035 PMC10327443

[17] A. González-Rueda, V. Pedrosa, R. C. Feord, C. Clopath, O. Paulsen, Activity-dependent downscaling of subthreshold synaptic inputs during slow-wave-sleep-like activity in vivo. Neuron 97, 1244–1252.e5 (2018). 29503184 10.1016/j.neuron.2018.01.047 PMC5873548

[18] D. F. English, S. McKenzie, T. Evans, K. Kim, E. Yoon, G. Buzsáki, Pyramidal cell-interneuron circuit architecture and dynamics in hippocampal networks. Neuron 96, 505–520.e7 (2017). 29024669 10.1016/j.neuron.2017.09.033 PMC5659748

[19] I. Gridchyn, P. Schoenenberger, J. O’Neill, J. Csicsvari, Optogenetic inhibition-mediated activity-dependent modification of CA1 pyramidal-interneuron connections during behavior. eLife 9, e61106 (2020). 33016875 10.7554/eLife.61106 PMC7575322

[20] A. Levi, L. Spivak, H. E. Sloin, S. Someck, E. Stark, Error correction and improved precision of spike timing in converging cortical networks. Cell Rep. 40, 111383 (2022). 36130516 10.1016/j.celrep.2022.111383 PMC9513803

[21] S. McKenzie, R. Huszár, D. F. English, K. Kim, F. Christensen, E. Yoon, G. Buzsáki, Preexisting hippocampal network dynamics constrain optogenetically induced place fields. Neuron 109, 1040–1054.e7 (2021). 33539763 10.1016/j.neuron.2021.01.011 PMC8095399

[22] X. Zhao, Y. Wang, N. Spruston, J. C. Magee, Membrane potential dynamics underlying context-dependent sensory responses in the hippocampus. Nat. Neurosci. 23, 881–891 (2020). 32451487 10.1038/s41593-020-0646-2

[23] L. L. McMahon, J. A. Kauer, Hippocampal interneurons express a novel form of synaptic plasticity. Neuron 18, 295–305 (1997). 9052799 10.1016/s0896-6273(00)80269-x

[24] A. I. Cowan, C. Stricker, L. J. Reece, S. J. Redman, Long-term plasticity at excitatory synapses on aspinous interneurons in area CA1 lacks synaptic specificity. J. Neurophysiol. 79, 13–20 (1998). 9425172 10.1152/jn.1998.79.1.13

[25] S. Royer, D. Paré, Conservation of total synaptic weight through balanced synaptic depression and potentiation. Nature 422, 518–522 (2003). 12673250 10.1038/nature01530

[26] J. H. Goldberg, R. Yuste, G. Tamas, Ca2^+^ imaging of mouse neocortical interneurone dendrites: Contribution of Ca2^+^-permeable AMPA and NMDA receptors to subthreshold Ca2^+^dynamics. J. Physiol. 551, 67–78 (2003). 12844507 10.1113/jphysiol.2003.042598 PMC2343145

[27] O. Camiré, L. Topolnik, Dendritic calcium nonlinearities switch the direction of synaptic plasticity in fast-spiking interneurons. J. Neurosci. 34, 3864–3877 (2014). 24623765 10.1523/JNEUROSCI.2253-13.2014 PMC6705275

[28] U. R. Karmarkar, D. V. Buonomano, Different forms of homeostatic plasticity are engaged with distinct temporal profiles. Eur. J. Neurosci. 23, 1575–1584 (2006). 16553621 10.1111/j.1460-9568.2006.04692.x

[29] A. Frick, J. Magee, D. Johnston, LTP is accompanied by an enhanced local excitability of pyramidal neuron dendrites. Nat. Neurosci. 7, 126–135 (2004). 14730307 10.1038/nn1178

[30] T. Alejandre-García, S. Kim, J. Pérez-Ortega, R. Yuste, Intrinsic excitability mechanisms of neuronal ensemble formation. eLife 11, e77470 (2022). 35506662 10.7554/eLife.77470 PMC9197391

[31] J. S. Lee, J. J. Briguglio, J. D. Cohen, S. Romani, A. K. Lee, The statistical structure of the hippocampal code for space as a function of time, context, and value. Cell 183, 620–635 (2020). 33035454 10.1016/j.cell.2020.09.024

[32] E. Stark, T. Koos, G. Buzsáki, Diode probes for spatiotemporal optical control of multiple neurons in freely moving animals. J. Neurophysiol. 108, 349–363 (2012). 22496529 10.1152/jn.00153.2012 PMC3434617

[33] E. Stark, R. Eichler, L. Roux, S. Fujisawa, H. G. Rotstein, G. Buzsáki, Inhibition-induced theta resonance in cortical circuits. Neuron 80, 1263–1276 (2013). 24314731 10.1016/j.neuron.2013.09.033 PMC3857586

[34] L. Spivak, A. Levi, H. E. Sloin, S. Someck, E. Stark, Deconvolution improves the detection and quantification of spike transmission gain from spike trains. Commun. Biol. 5, 520 (2022). 35641587 10.1038/s42003-022-03450-5 PMC9156687

[35] D. Dupret, J. O’Neill, J. Csicsvari, Dynamic reconfiguration of hippocampal interneuron circuits during spatial learning. Neuron 78, 166–180 (2013). 23523593 10.1016/j.neuron.2013.01.033 PMC3627224

[36] M. Chistiakova, V. Ilin, M. Roshchin, N. Bannon, A. Malyshev, Z. Kisvárday, M. Volgushev, Distinct heterosynaptic plasticity in fast spiking and non-fast-spiking inhibitory neurons in rat visual cortex. J. Neurosci. 39, 6865–6878 (2019). 31300522 10.1523/JNEUROSCI.3039-18.2019 PMC6733570

[37] S. V. Rolotti, M. S. Ahmed, M. Szoboszlay, T. Geiller, A. Negrean, H. Blockus, K. C. Gonzalez, F. T. Sparks, A. S. Solis Canales, A. L. Tuttman, D. S. Peterka, B. V. Zemelman, F. Polleux, A. Losonczy, Local feedback inhibition tightly controls rapid formation of hippocampal place fields. Neuron 110, 783–794.e6 (2022). 34990571 10.1016/j.neuron.2021.12.003 PMC8897257

[38] P. Rao-Ruiz, J. Yu, S. A. Kushner, S. A. Josselyn, Neuronal competition: Microcircuit mechanisms define the sparsity of the engram. Curr. Opin. Neurobiol. 54, 163–170 (2019). 30423499 10.1016/j.conb.2018.10.013 PMC9730430

[39] S. Fujisawa, A. Amarasingham, M. T. Harrison, G. Buzsáki, Behavior-dependent short-term assembly dynamics in the medial prefrontal cortex. Nat. Neurosci. 11, 823–833 (2008). 18516033 10.1038/nn.2134 PMC2562676

[40] D. M. Kullmann, K. P. Lamsa, LTP and LTD in cortical GABAergic interneurons: Emerging rules and roles. Neuropharmacology 60, 712–719 (2011). 21185319 10.1016/j.neuropharm.2010.12.020

[41] P. Y.-P. Lau, L. Katona, P. Saghy, K. Newton, P. Somogyi, K. P. Lamsa, Long-term plasticity in identified hippocampal GABAergic interneurons in the CA1 area in vivo. Brain Struct. Funct. 222, 1809–1827 (2017). 27783219 10.1007/s00429-016-1309-7 PMC5406446

[42] E.-L. Yap, N. L. Pettit, C. P. Davis, M. A. Nagy, D. A. Harmin, E. Golden, O. Dagliyan, C. Lin, S. Rudolph, N. Sharma, E. C. Griffith, C. D. Harvey, M. E. Greenberg, Bidirectional perisomatic inhibitory plasticity of a Fos neuronal network. Nature 590, 115–121 (2021). 33299180 10.1038/s41586-020-3031-0 PMC7864877

[43] S.-H. Lee, I. Marchionni, M. Bezaire, C. Varga, N. Danielson, M. Lovett-Barron, A. Losonczy, I. Soltesz, Parvalbumin-positive basket cells differentiate among hippocampal pyramidal cells. Neuron 82, 1129–1144 (2014). 24836505 10.1016/j.neuron.2014.03.034 PMC4076442

[44] A. Navas-Olive, M. Valero, T. Jurado-Parras, A. de Salas-Quiroga, R. G. Averkin, G. Gambino, E. Cid, L. M. de la Prida, Multimodal determinants of phase-locked dynamics across deep-superficial hippocampal sublayers during theta oscillations. Nat. Commun. 11, 2217 (2020). 32371879 10.1038/s41467-020-15840-6 PMC7200700

[45] R. Huszár, Y. Zhang, H. Blockus, G. Buzsáki, Preconfigured dynamics in the hippocampus are guided by embryonic birthdate and rate of neurogenesis. Nat. Neurosci. 25, 1201–1212 (2022). 35995878 10.1038/s41593-022-01138-x PMC10807234

[46] M. Abeles, *Corticonics: Neural Circuits of the Cerebral Cortex* (Cambridge Univ. Press, 1991).

[47] E. Bienenstock, A model of neocortex. Netw. Comput. Neural Syst. 6, 179–224 (1995).

[48] G. Buzsáki, Neural syntax: Cell assemblies, synapsembles, and readers. Neuron 68, 362–385 (2010). 21040841 10.1016/j.neuron.2010.09.023 PMC3005627

[49] D. O. Hebb, *The Organization of Behavior: A Neuropsychological Theory* (Psychology Press, 1949).

[50] J. J. Hopfield, Neural networks and physical systems with emergent collective computational abilities. Proc. Natl. Acad. Sci. *U.S.A.* 79, 2554–2558 (1982). 6953413 10.1073/pnas.79.8.2554 PMC346238

[51] F. Gambino, A. Holtmaat, Spike-timing-dependent potentiation of sensory surround in the somatosensory cortex is facilitated by deprivation-mediated disinhibition. Neuron 75, 490–502 (2012). 22884332 10.1016/j.neuron.2012.05.020

[52] W. Zhang, D. J. Linden, The other side of the engram: Experience-driven changes in neuronal intrinsic excitability. Nat. Rev. Neurosci. 4, 885–900 (2003). 14595400 10.1038/nrn1248

[53] J. H. Goldberg, G. Tamas, D. Aronov, R. Yuste, Calcium microdomains in aspiny dendrites. Neuron 40, 807–821 (2003). 14622584 10.1016/s0896-6273(03)00714-1

[54] B. Rozsa, T. Zelles, E. S. Vizi, B. Lendvai, Distance-dependent scaling of calcium transients evoked by backpropagating spikes and synaptic activity in dendrites of hippocampal interneurons. J. Neurosci. 24, 661–670 (2004). 14736852 10.1523/JNEUROSCI.3906-03.2004 PMC6729270

[55] B. Chiovini, G. F. Turi, G. Katona, A. Kaszás, D. Pálfi, P. Maák, G. Szalay, M. F. Szabó, G. Szabó, Z. Szadai, S. Káli, B. Rózsa, Dendritic spikes induce ripples in parvalbumin interneurons during hippocampal sharp waves. Neuron 82, 908–924 (2014). 24853946 10.1016/j.neuron.2014.04.004

[56] F. Laezza, J. J. Doherty, R. Dingledine, Long-term depression in hippocampal interneurons: Joint requirement for pre- and postsynaptic events. Science 285, 1411–1414 (1999). 10464102 10.1126/science.285.5432.1411

[57] K. P. Lamsa, J. H. Heeroma, P. Somogyi, D. A. Rusakov, D. M. Kullmann, Anti-Hebbian long-term potentiation in the hippocampal feedback inhibitory circuit. Science 315, 1262–1266 (2007). 17332410 10.1126/science.1137450 PMC3369266

[58] T. Hainmüller, K. Krieglstein, A. Kulik, M. Bartos, Joint CP-AMPA and group I mGlu receptor activation is required for synaptic plasticity in dentate gyrus fast-spiking interneurons. Proc. Natl. Acad. Sci. U.S.A. 111, 13211–13216 (2014). 25161282 10.1073/pnas.1409394111 PMC4246940

[59] N. M. Bannon, M. Chistiakova, M. Volgushev, Synaptic plasticity in cortical inhibitory neurons: What mechanisms may help to balance synaptic weight changes? Front. Cell. Neurosci. 14, 204 (2020). 33100968 10.3389/fncel.2020.00204 PMC7500144

[60] Y. Perez, F. Morin, J.-C. Lacaille, A Hebbian form of long-term potentiation dependent on mGluR1a in hippocampal inhibitory interneurons. Proc. Natl. Acad. Sci. U.S.A. 98, 9401–9406 (2001). 11447296 10.1073/pnas.161493498 PMC55433

[61] S. Huang, R. L. Huganir, A. Kirkwood, Adrenergic gating of Hebbian spike-timing-dependent plasticity in cortical interneurons. J. Neurosci. 33, 13171–13178 (2013). 23926270 10.1523/JNEUROSCI.5741-12.2013 PMC3735889

[62] W. Nissen, A. Szabo, J. Somogyi, P. Somogyi, K. P. Lamsa, Cell type-specific long-term plasticity at glutamatergic synapses onto hippocampal interneurons expressing either parvalbumin or CB _1_ cannabinoid receptor. J. Neurosci. 30, 1337–1347 (2010). 20107060 10.1523/JNEUROSCI.3481-09.2010 PMC2817897

[63] R. K. Mishra, S. Kim, S. J. Guzman, P. Jonas, Symmetric spike timing-dependent plasticity at CA3–CA3 synapses optimizes storage and recall in autoassociative networks. Nat. Commun. 7, 11552 (2016). 27174042 10.1038/ncomms11552 PMC4869174

[64] H. Yao, Y. Shen, Y. Dan, Intracortical mechanism of stimulus-timing-dependent plasticity in visual cortical orientation tuning. Proc. Natl. Acad. Sci. *U.S.A.* 101, 5081–5086 (2004). 15044699 10.1073/pnas.0302510101 PMC387377

[65] S. Cassenaer, G. Laurent, Conditional modulation of spike-timing-dependent plasticity for olfactory learning. Nature 482, 47–52 (2012). 22278062 10.1038/nature10776

[66] P. Dayan, L. F. Abbott, *Theoretical Neuroscience: Computational and Mathematical Modeling of Neural Systems* (MIT Press, 2001).

[67] C. Sun, Q. Chen, K. Chen, G. He, Y. Fu, L. Li, Unsupervised learning based on temporal coding using STDP in spiking neural networks, in *2022 IEEE International Symposium on Circuits and Systems (ISCAS)* (2022), Austin, TX, 27 May to 1 June 2022, pp. 2142–2146.

[68] K. C. Bittner, A. D. Milstein, C. Grienberger, S. Romani, J. C. Magee, Behavioral time scale synaptic plasticity underlies CA1 place fields. Science 357, 1033–1036 (2017). 28883072 10.1126/science.aan3846 PMC7289271

[69] K. C. Bittner, C. Grienberger, S. P. Vaidya, A. D. Milstein, J. J. Macklin, J. Suh, S. Tonegawa, J. C. Magee, Conjunctive input processing drives feature selectivity in hippocampal CA1 neurons. Nat. Neurosci. 18, 1133–1142 (2015). 26167906 10.1038/nn.4062 PMC4888374

[70] J. K. O’Hare, K. C. Gonzalez, S. A. Herrlinger, Y. Hirabayashi, V. L. Hewitt, H. Blockus, M. Szoboszlay, S. V. Rolotti, T. C. Geiller, A. Negrean, V. Chelur, F. Polleux, A. Losonczy, Compartment-specific tuning of dendritic feature selectivity by intracellular Ca ^2+^ release. Science 375, eabm1670 (2022). 10.1126/science.abm1670 PMC9667905 35298275

[71] G. Dragoi, G. Buzsáki, Temporal encoding of place sequences by hippocampal cell assemblies. Neuron 50, 145–157 (2006). 16600862 10.1016/j.neuron.2006.02.023

[72] J. O’Keefe, M. L. Recce, Phase relationship between hippocampal place units and the EEG theta rhythm. Hippocampus 3, 317–330 (1993). 8353611 10.1002/hipo.450030307

[73] H. E. Sloin, A. Levi, S. Someck, L. Spivak, E. Stark, High fidelity theta phase rolling of CA1 neurons. J. Neurosci. 42, 3184–3196 (2022). 35264413 10.1523/JNEUROSCI.2151-21.2022 PMC8994535

[74] E. Stark, L. Roux, R. Eichler, G. Buzsáki, Local generation of multineuronal spike sequences in the hippocampal CA1 region. Proc. Natl. Acad. Sci. *U.S.A.* 112, 10521–10526 (2015). 10.1073/pnas.1508785112 PMC4547251 26240336

[75] M. Wang, D. J. Foster, B. E. Pfeiffer, Alternating sequences of future and past behavior encoded within hippocampal theta oscillations. Science 370, 247–250 (2020). 33033222 10.1126/science.abb4151 PMC8594055

[76] O. Noked, A. Levi, S. Someck, O. Amber-Vitos, E. Stark, Bidirectional optogenetic control of inhibitory neurons in freely-moving mice. IEEE Trans. Biomed. Eng. 68, 416–427 (2021). 32746022 10.1109/TBME.2020.3001242

[77] N. Gaspar, R. Eichler, E. Stark, A novel low-noise movement tracking system with real-time analog output for closed-loop experiments. J. Neurosci. Methods 318, 69–77 (2019). 30650336 10.1016/j.jneumeth.2018.12.016

[78] C. Rossant, S. N. Kadir, D. F. M. Goodman, J. Schulman, M. L. D. Hunter, A. B. Saleem, A. Grosmark, M. Belluscio, G. H. Denfield, A. S. Ecker, A. S. Tolias, S. Solomon, G. Buzsáki, M. Carandini, K. D. Harris, Spike sorting for large, dense electrode arrays. Nat. Neurosci. 19, 634–641 (2016). 26974951 10.1038/nn.4268 PMC4817237

[79] M. Pachitariu, N. Steinmetz, S. Kadir, M. Carandini, K. D. Harris, Kilosort: Realtime spike-sorting for extracellular electrophysiology with hundreds of channels. bioRxiv 061481 [Preprint] (2016). 10.1101/061481.

[80] E. Stark, M. Abeles, Unbiased estimation of precise temporal correlations between spike trains. J. Neurosci. Methods 179, 90–100 (2009). 19167428 10.1016/j.jneumeth.2008.12.029

