# Supplementary material for: Wired together, change together: Spike timing modifies transmission in converging assemblies
Source: Sci Adv. 2024 Jan 17;10(3):eadj4411. doi: 10.1126/sciadv.adj4411 (PMC10793958; doi:10.1126/sciadv.adj4411)
Supplement: Supplementary file 1 — Figs. S1 to S8 Tables S1 to S3 [file sciadv.adj4411_sm.pdf]

Science Advances
AAAS

Supplementary Materials for

Wired together, change together: Spike timing modifies transmission in converging assemblies

Lidor Spivak *et al.*

Corresponding author: Eran Stark, eranstark@sci.haifa.ac.il

Sci. Adv. **10**, eadj4411 (2024)
DOI: 10.1126/sciadv.adj4411

This PDF file includes:

Figs. S1 to S8
Tables S1 to S3

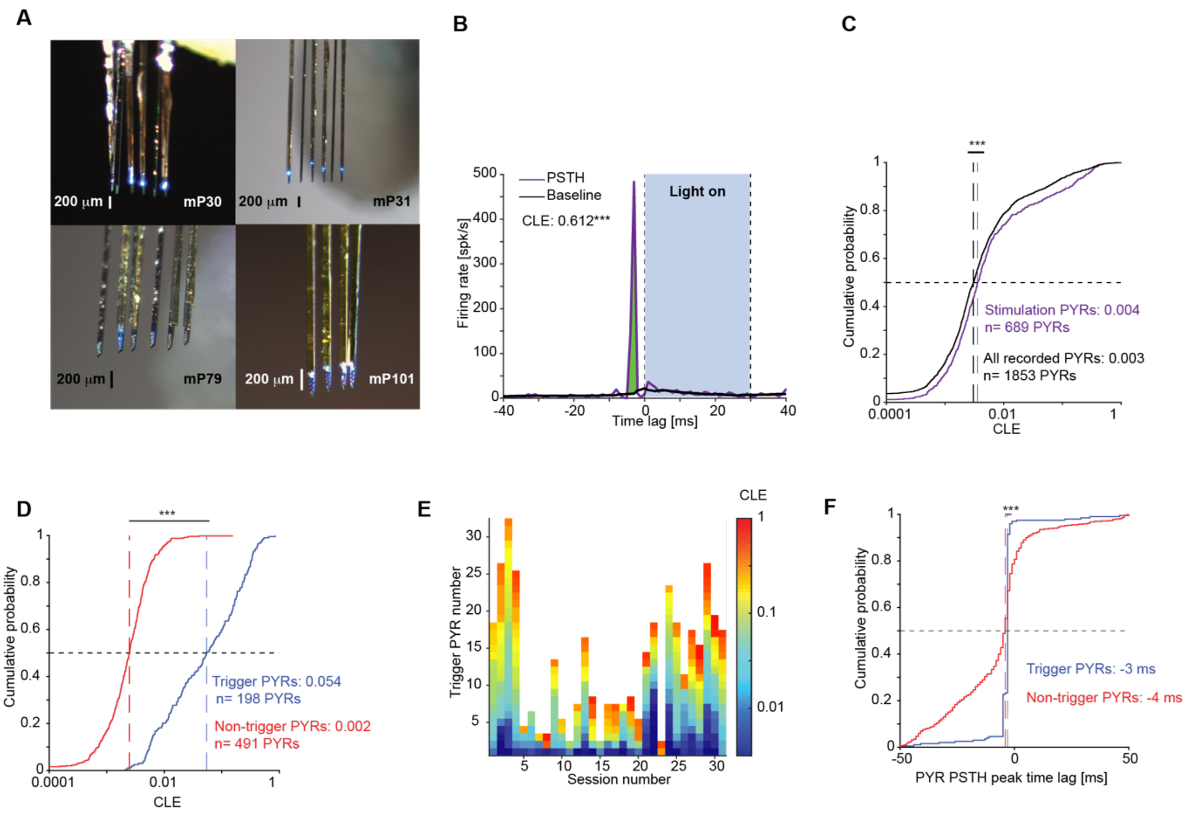

Fig. S1. PYR spikes trigger closed-loop light stimulation with millisecond timescale resolution. (**A**) Photos of optical fibers attached to the four silicon probes which were implanted in PV::ChR2 mice and employed for closed-loop stimulation experiments (**table S2**). (**B**) An example of a PSTH from a trigger PYR with high CLE. The CLE is calculated as the area under the peak (green) in the $-5 \leq \tau < 0$ ms ROI, and the baseline activity is determined by hollow median filtering (5 ms halfwidth) of the PSTH (black line). ***: $p<0.001$, Poisson test. (**C**) Distribution of CLE values for all PYRs recorded during Stimulation sessions (black) and for the PYRs participating in Stimulation pairs as shown in **table S2**. Here, in **D**, and in **F**, ***: $p<0.001$, U-test. (**D**) Distributions of CLE values for the Stimulation PYRs. PYRs which exhibit a consistent PSTH peak within the closed-loop ROI ($p<0.001$ Poisson test) are defined as “trigger” PYRs. (**E**) The number of trigger PYRs in every session. The color represents the CLE of each PYR. Sessions are sorted chronologically. (**F**) Distribution of PSTH peak time lags for trigger (n=198) and non-trigger PYRs (n=491). SDs are 8 ms for trigger PYRs and larger (14 ms) for non-trigger PYRs ($p<0.001$, permutation test).

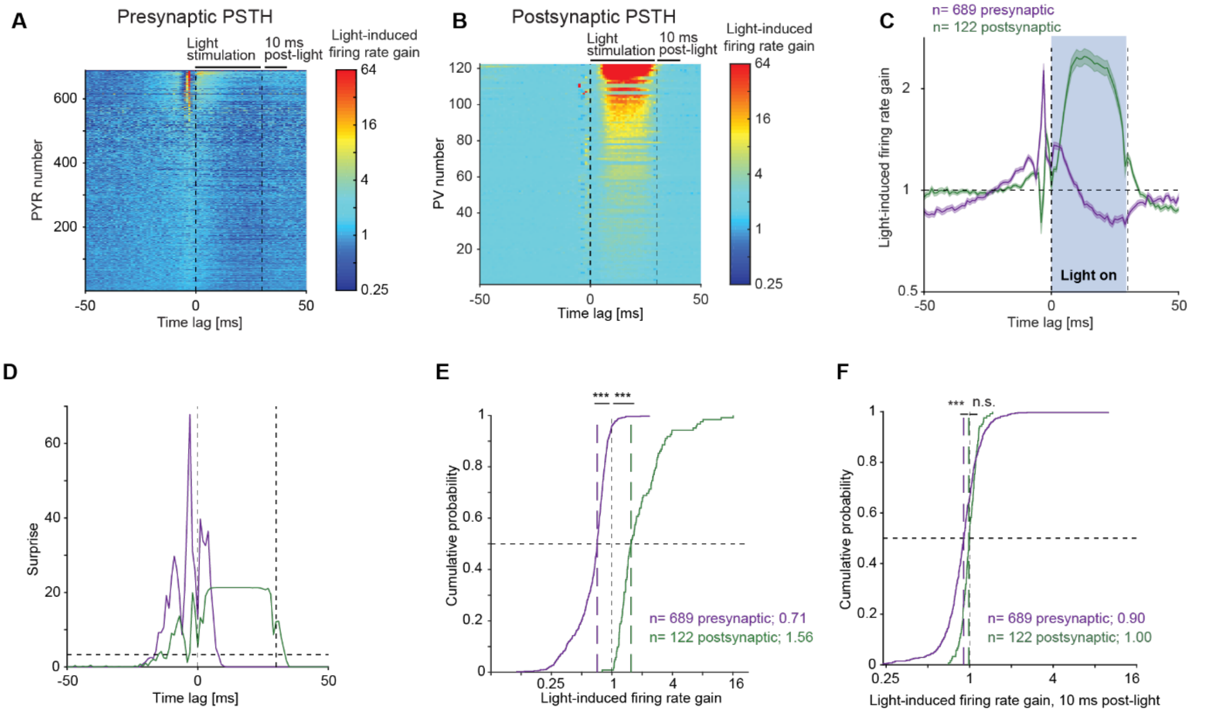

Fig. S2. Rate changes during closed-loop stimulation. (**A**) Gain PSTHs of 689 PYRs during closed-loop stimulation, with zero time lag indicating light onset (as in **Fig. 3A**). Every gain PSTH is scaled by the baseline firing rate, estimated for every unit during a 15 ms period starting 30 ms before light onset and ending 15 ms before light onset. (**B**) Same as **A**, for the 122 PV cells. (**C**) Mean light-induced firing rate gain of all 689 PYRs (purple) and 122 PV cells (green). Error bands, SEM. (**D**) Surprise, defined as the base-10 logarithm of the p-value estimating the probability that the median gain of PYRs and PVs exceeds unity for each 1 ms PSTH bin (Wilcoxon's signed-rank test comparing to unity gain null). The dashed line corresponds to the Bonferroni-corrected chance level (0.05/101). (**E**) Distribution of the light-induced firing rate gain for the n=689/122 PYR/PV cells during light stimulation. Here and in **F**, n.s./***: $p>0.05/p<0.001$, Wilcoxon's signed-rank test compared to a unity gain null. (**F**) Distribution of the post-light gain, computed as the ratio between the mean firing rates during the 10 ms post-light and the baseline firing rates.

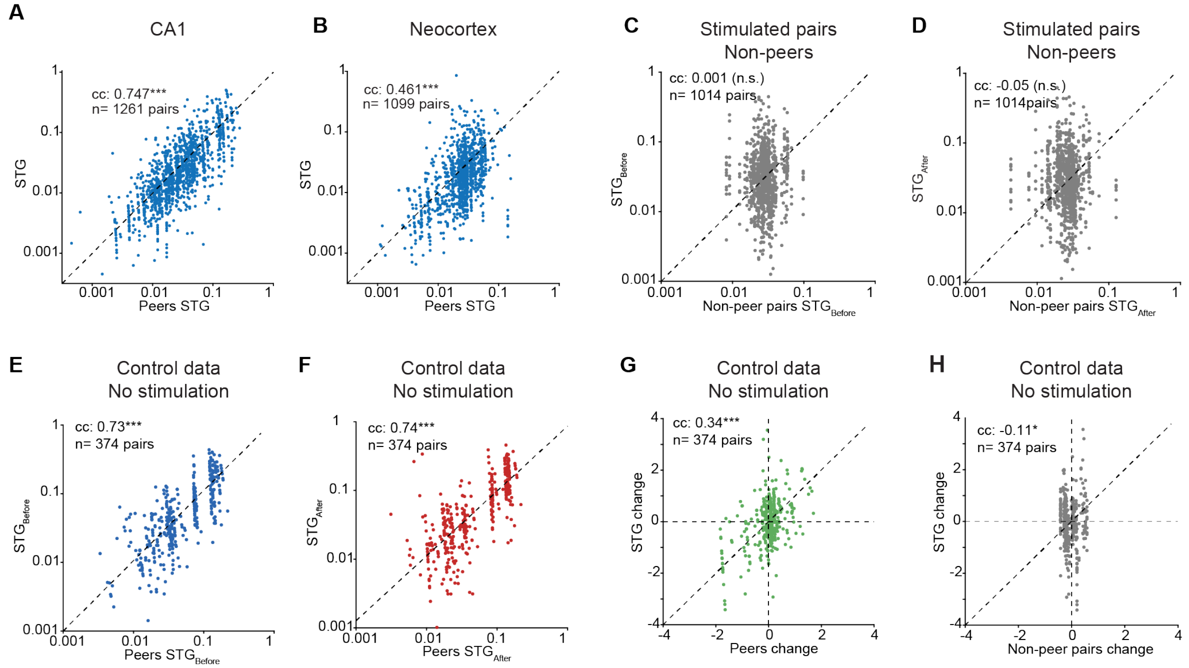

Fig. S3. STGs are similar for pairs in the same converging assembly. (**A**) STG of every CA1 PYR-interneurons pair vs. mean STG of all peers. Peers, PYR-interneuron pairs that share the same postsynaptic interneuron, forming a CA. n=1261 pairs in 139 CAs recorded from hippocampal region CA1 of three mice(*34*). Here and in **B-H**, n.s./*/**/***: $p>0.05/p<0.05/p<0.01/p<0.001$, permutation test. (**B**) STGs of neocortical PYR-interneuron pairs vs. mean peers STG. n=1099 pairs in 100 CAs recorded from six mice(*20*). (**C**) The STG_{Before} of every pair vs. the mean STG_{Before} of all non-peer pairs. Here and in **D**, data are shown for the stimulated 1014/1026 PYR-PV pairs as in **Fig. 2C**. (**D**) Same as **C** for the STG_{After}. (**E**) The STG_{Before} of every pair vs. the mean STG_{Before} of all peers. Here and in **F-H**, data are shown for the 374/388 PYR-PV Control pairs which were part of a CA with more than one presynaptic PYR, and did not undergo light stimulation during the “Experience” epoch. (**F**) Same as **E** for the STG_{After}. (**G**) STG change vs. the mean STG change of all peers. (**H**) Same as **G** for non-peer pairs.

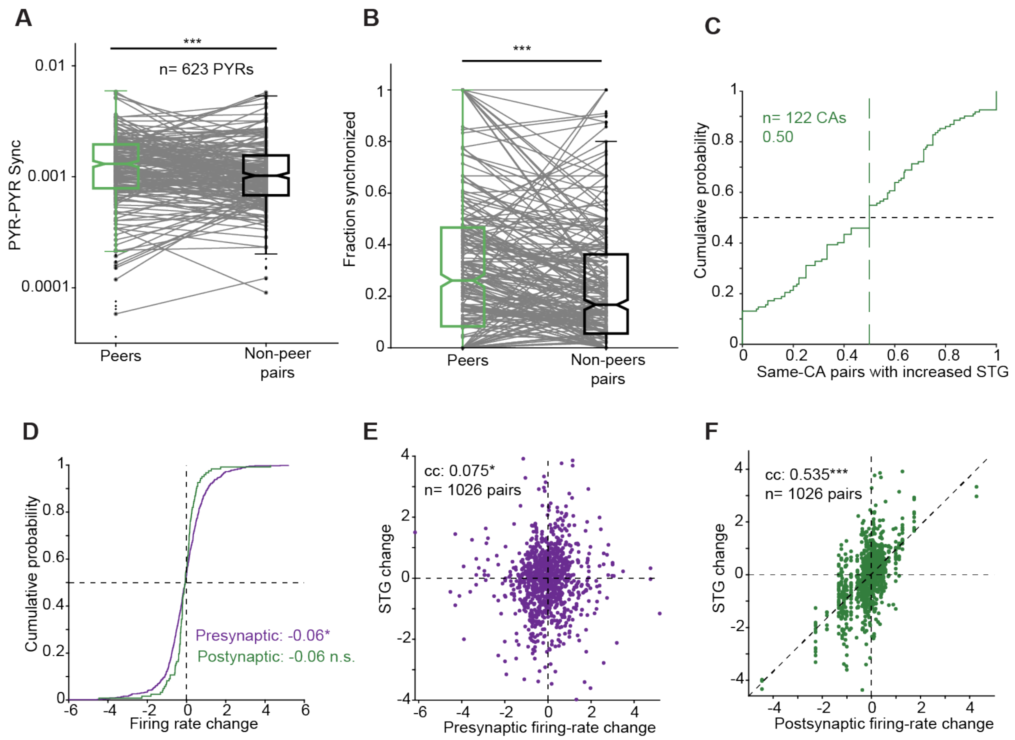

Fig. S4. PYRs which belong to the same converging assembly are synchronized. (**A**) Mean synchronous firing between a single PYR and either peer PYRs or non-peer PYRs. n=623/689 PYRs which have both peers and non-peers PYR recorded on a different shank. Here and in **B**, ***: p<0.001, Wilcoxon's paired test. (**B**) Same as **A** for the fraction of synchronized peers and synchronized non-peers of each PYR. (**C**) Distribution of the fraction of pairs that exhibit an STG increase in every CA. (**D**) Distribution of the firing rate change for PYRs (n=689) and PV cells (n=122). The firing rate change is defined as the base-2 logarithm of the ratio between the firing rate during the After epoch and the firing rate during the Before epoch. Median [IQR] values are -0.06 [-0.61 0.47] for the PYRs, and -0.06 [-0.32 0.18] for the PV cells. n.s./*: p>0.05/p<0.05, Wilcoxon's test comparing to a zero-change null. (**E-F**) STG changes vs. firing rate changes of the presynaptic or the postsynaptic cell.

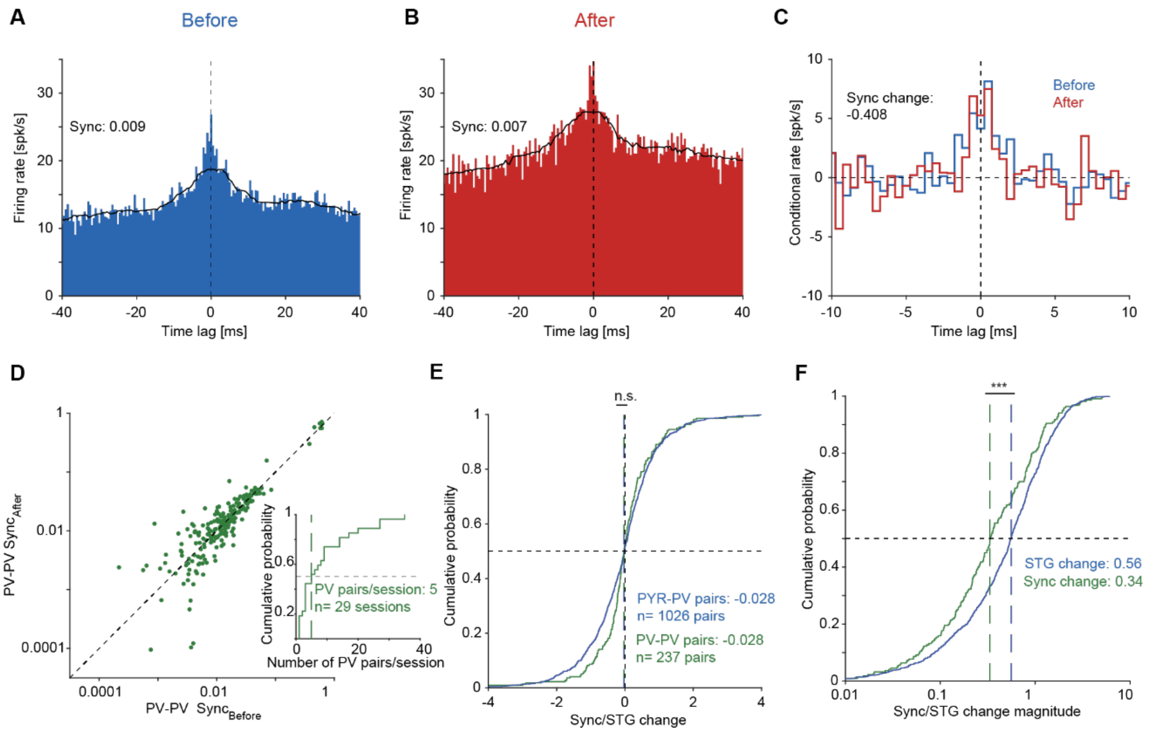

Fig. S5. Changes in PV synchronous firing are smaller than changes in PYR-PV transmission. (**A-B**) CCHs between two simultaneously recorded PV cells during the Before (**A**) and the After (**B**) epochs. (**C**) Overlaid conditional rate histograms for the no-light epochs, obtained by removing the baseline activity. (**D**) Synchrony of PV pairs during the After epoch vs. the Before epoch. **Inset**, Distribution of the number of simultaneously recorded of PV pairs. PV pairs recorded on the same shank are not included. (**E**) Distribution of the PYR-PV STG change (blue) as in **Fig. 1L**, and the PV-PV Sync change computed from the data shown in **D**. Here and in **F**, n.s./***: $p>0.05$/$p<0.001$, U-test. (**F**) Magnitude (absolute value) of STG changes (blue) as in **Fig. 1M**, and magnitude of PV-PV Sync.

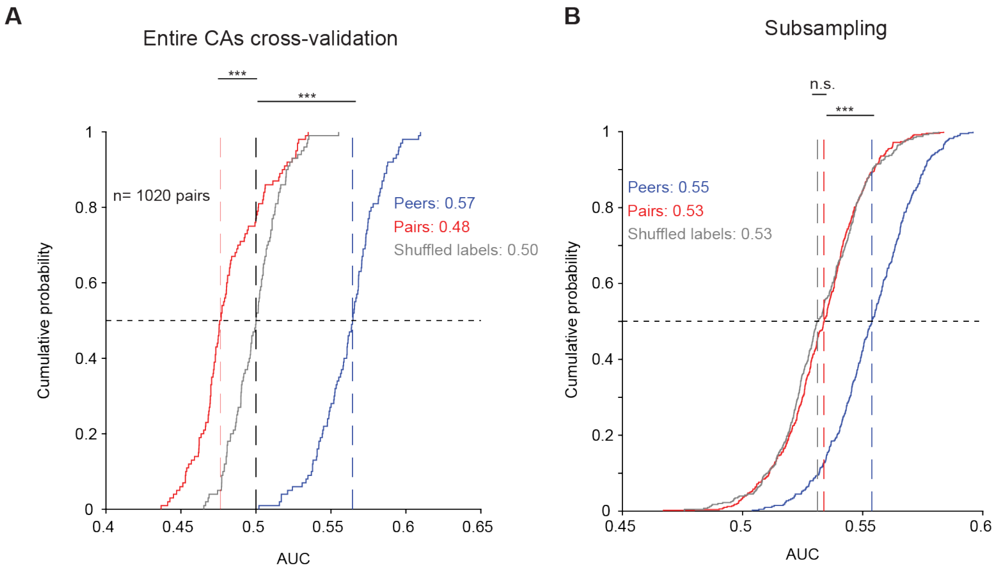

Fig. S6. Pairwise STG changes can be predicted more accurately by peer pairs spike timing even when assembly structure information is removed. (**A**) For removing all assembly-based information from the classification process, we utilized two approaches. In the first approach, we trained cross-validated classifiers with carefully-constructed cross-validation folds. Specifically, each fold in the training and test sets utilized only entire assemblies, ensuring that pairs belonging to the same assembly were never used in both the training and test sets. We trained three types of five-fold cross-validated linear SVMs: pairs, peer pairs, and peers with shuffled labels. The process was repeated 100 times, and every iteration employed random partitions which were composed only from entire assemblies. Here and in **B**, n.s./***: $p>0.05$/$p<0.001$, U-test. (**B**) As an alternative approach for removing all assembly-based information from the classification process, we sampled only one pair from every CA. We trained three five-fold cross-validated linear SVMs: pairs, peer pairs, and peers with shuffled labels. Every classifier was trained during 100 independent runs, and in every run a random subset of 116/1020 pairs, one from every CAs, was employed.

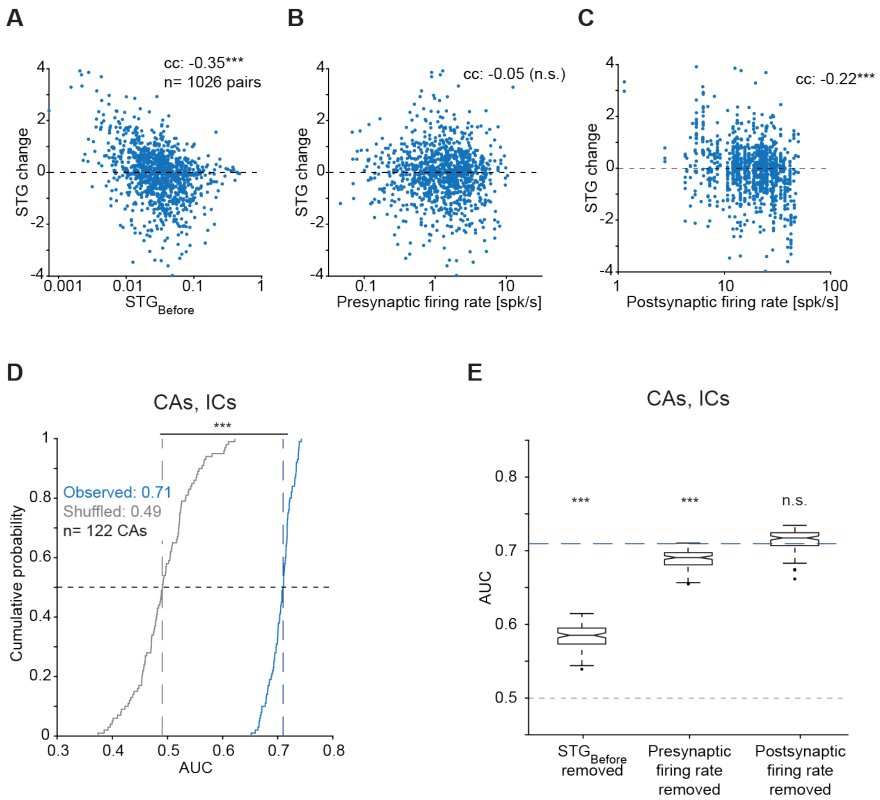

Fig. S7. Initial conditions carry information about CA STG changes. (**A-C**) Pairwise STG change vs. three initial conditions (ICs) derived from the Before epoch. n.s./***: p>0.05/p<0.001, permutation test. (**A**) STG_{Before}. (**B**) The mean firing rate of the presynaptic PYR. (**C**) The mean firing rate of the postsynaptic PV cell. (**D**) AUCs produced by cross-validated binary classifiers (linear SVMs) trained with all three mean CA ICs features shown in **Fig. 4E**. ***: p<0.001, U-test. (**E**) AUCs for three classifiers. For every classifier, a different feature was removed. Top dashed line, median AUC of the full model. n.s./***: p>0.05/p<0.001, Wilcoxon's test.

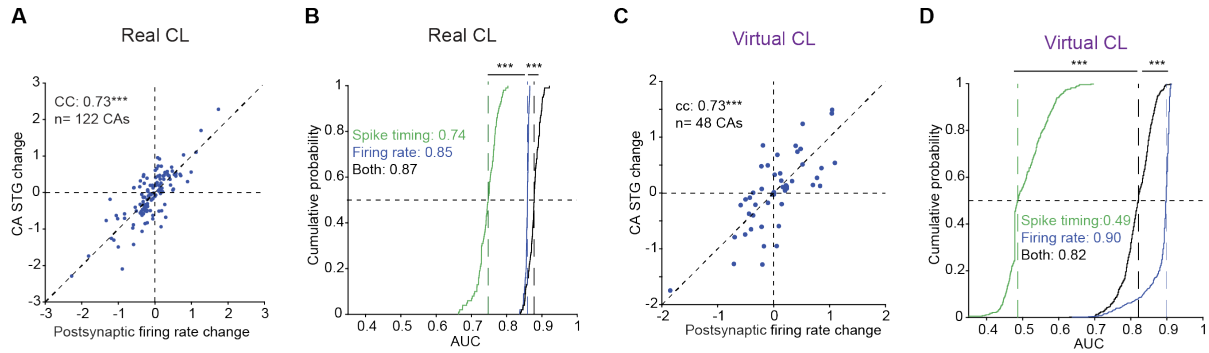

Fig. S8. Spike timing in the Control dataset does not contain information about STG changes. (**A**) CA mean STG changes vs. the postsynaptic firing rate changes for the real closed-loop (CL) dataset. Here, the postsynaptic firing rate change is defined as the base-2 logarithm of the ratio between the PV firing rate during the After and Before epochs. Here and in **C**, ***: p<0.001, permutation test. (**B**) AUCs for the three different classifiers using the real CL dataset (as in **Fig. 5D**). Here and in **D**, ***: p<0.001, U-test. In the real CL dataset, considering spike timing improves the prediction. (**C**) CA mean STG change vs. the PV firing rate changes for the virtual CL dataset. (**D**) AUC distributions for the three different classifiers in the virtual CL dataset (as in **Fig. 5L**). In the virtual CL dataset, considering spike timing deteriorates the prediction.

Tables

Table S1. List of experimental animals

Animal ID	Sex	Age[1] [week]	Weight[1] [g]	Probe[2]	Sessions	PYRs	Interneurons	Pairs	Connected pairs[3]
mDS1	Male	14	25.7	DS64	5	175	25	710	268
mDS2	Male	30	24.2	DS64	1	49	15	735	237
mP101	Male	16	29.7	Buzsaki32	2	56	12	279	17
mP30	Male	14	28.6	Stark64	6	262	49	2371	484
mP31	Male	16	30	Stark64	12	516	140	5269	751
mP79	Male	17	30.1	DS128	12	1013	224	16587	2453
mS51	Male	8	22.7	Stark128	2	175	84	7775	445
**Total**					**40**	**2246**	**549**	**33726**	**4655**

[1] At the time of implantation.
[2] DS64, dual-sided64; DS128, dual-sided128.
[3] Pairs with excitatory monosynaptic connectivity ($p<0.001$, Poisson test).

Table S2. Units and pairs used in the closed-loop Stimulation analyses

Animal ID	Strain	All sessions	Valid[1] sessions	PYRs	PV cells[2]	Pairs	STG increase[3]	STG decrease[3]	CAs
mP101	PV::ChR2	1	1	9	3	10	4	2	2
mP30	PV::ChR2	6	6	98	20	142	12	19	19
mP31	PV::ChR2	12	10	163	33	210	19	21	31
mP79	PV::ChR2	12	12	419	66	664	81	118	64
**Total**		**31**	**29**	**689**	**122**	**1026**	**116**	**160**	**116**

[1] Sessions with at least one valid connected PYR-PV pair.

[2] Units with firing rate increase during closed-loop illumination ($p<0.05$, Poisson test).

[3] Pairs with consistent STG increase/decrease ($p<0.025$, permutation test).

Table S3. Units and pairs used in the Control and virtual closed-loop analyses

Animal ID	All sessions	Valid[1] sessions	PYRs	Interneurons	Pairs	STG increase[2]	STG decrease[2]	CAs
mDS1	5	4	52	9	83	5	8	6
mDS2	1	1	41	5	93	22	15	5
mP101	1	0	0	0	0	0	0	0
mP31	1	1	20	5	29	4	3	4
mP79	1	1	46	9	80	8	8	8
mS51	2	2	65	20	103	15	11	17
**Total**	**11**	**9**	**224**	**48**	**388**	**54**	**45**	**40**

[1] Sessions with at least one valid connected PYR-INT pair.

[2] Pairs with consistent STG increase/decrease ($p<0.025$, permutation test).
